# Supplementary material for: Intravenous immunoglobulin enhances intestinal stem cell regeneration to mitigate radiation-induced enteritis via promoting β-catenin nuclear translocation
Source: Stem Cell Reports. 2026 Jun 4;21(7):102934. doi: 10.1016/j.stemcr.2026.102934 (PMC13385420; doi:10.1016/j.stemcr.2026.102934)
Supplement: Document S1. Figures S1–S8 [file mmc1.pdf]

Stem Cell Reports, Volume *21*

Supplemental Information

Intravenous immunoglobulin enhances intestinal stem cell regeneration to mitigate radiation-induced enteritis via promoting β-catenin nuclear translocation

Jia He, Tiancheng Chu, Ping Fu, Peng Jiang, Li Ma, Fengjuan Liu, Xi Du, Zhenni Xu, Jun Xu, Lu Cheng, Changqing Li, Dengqun Liu, and Zongkui Wang

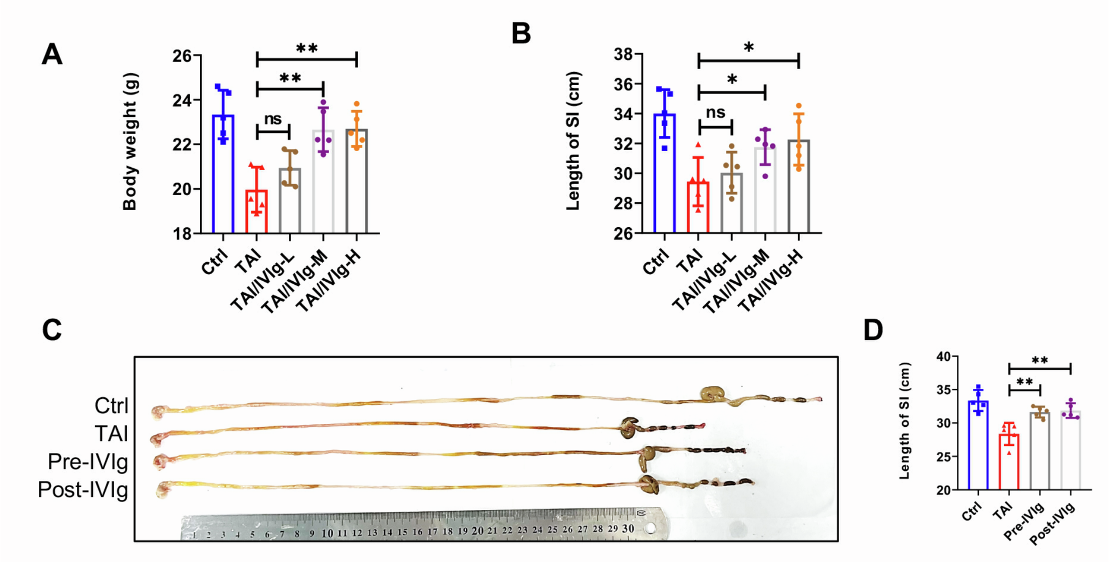

Figure S1. Effects of different IVIg concentrations and treatment timing on TAI mice. (A) Effects of different IVIg concentrations on body weight of TAI mice. (B) Effects of different IVIg concentrations on small intestinal length of TAI mice. (C) Effects of IVIg pretreatment and treatment on mouse intestinal tissue. (D) Statistical analysis of intestinal length. n = 5 mice. * $p < 0.05$, ** $p < 0.01$, *** $p < 0.001$, ns = not significant.

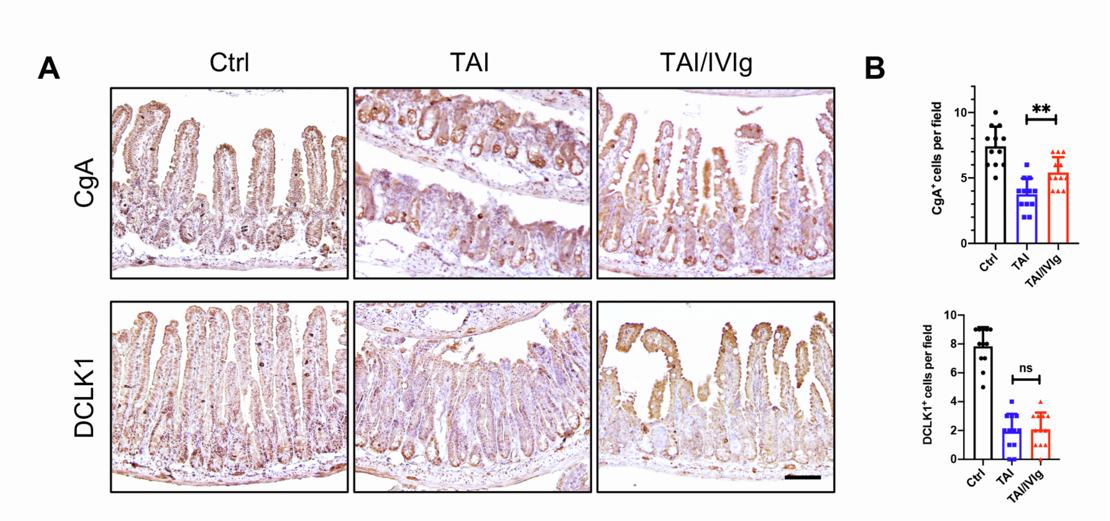

Figure S2. Effects of IVIg on other epithelial cells after TAI induction of RIE. (A) Representative images of CgA^{+} cells of small intestines (Bar = 100 μm). (B) Statistical analysis of CgA^{+} cells, n = 12 crypts. ** $p < 0.01$, ns = not significant.

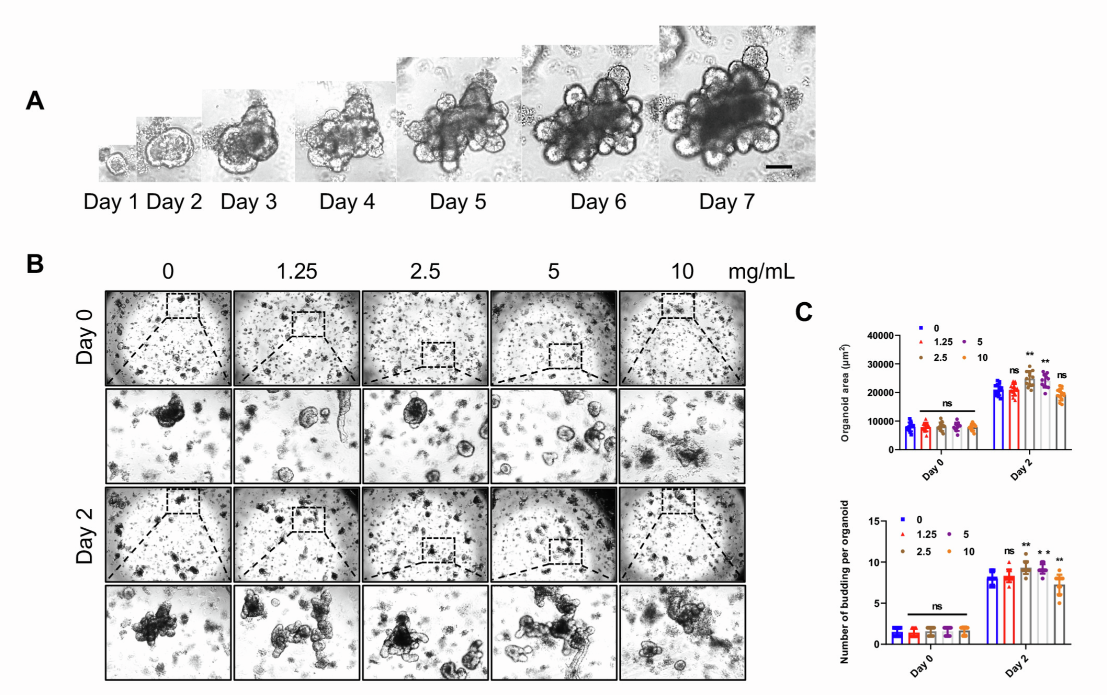

Figure S3. IVIg promoted the growth of healthy enteroids. (A) Morphological images of healthy intestinal enteroids under microscopy (Bar = 100 μm). (B) Microscopic images of intestinal enteroids after treatment with IVIg (Bar = 500 μm). (C) Quantitative analysis of the area and budding number per intestinal enteroid, n = 12 enteroids. ** $p < 0.01$, ns = not significant.

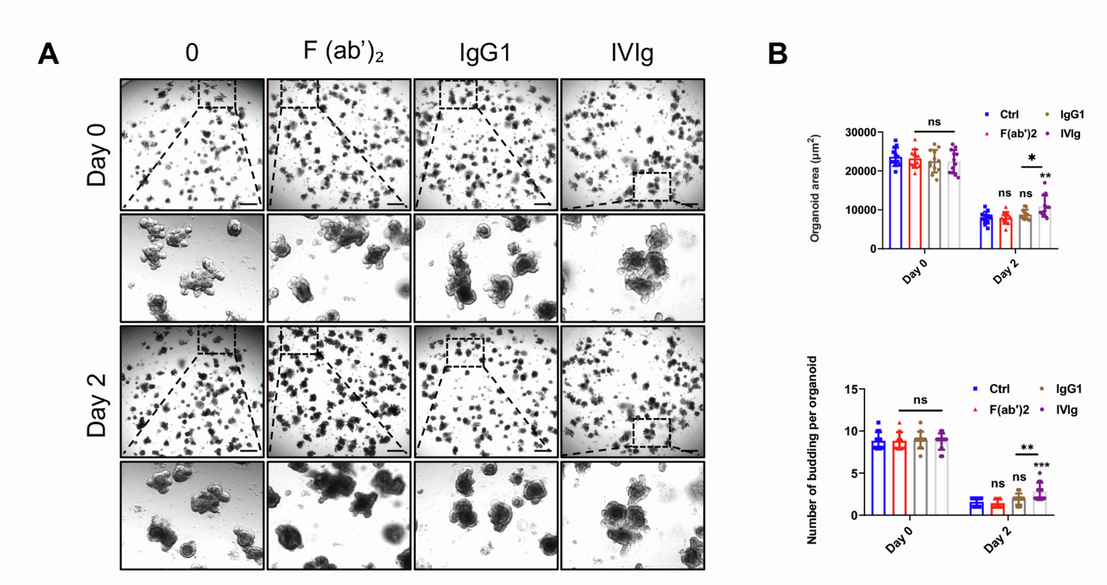

Figure S4. Effects of IVIg and its fragments on irradiated intestinal enteroids. (A) Representative microscopic images of irradiated intestinal enteroids treated with $F(ab')_2$, IgG1 or IVIg (Bar = 500 μm). (B) Quantitative analysis of the area and budding number per intestinal enteroid, n = 12 enteroids. * $p < 0.05$, ** $p < 0.01$, *** $p < 0.001$, ns = not significant.

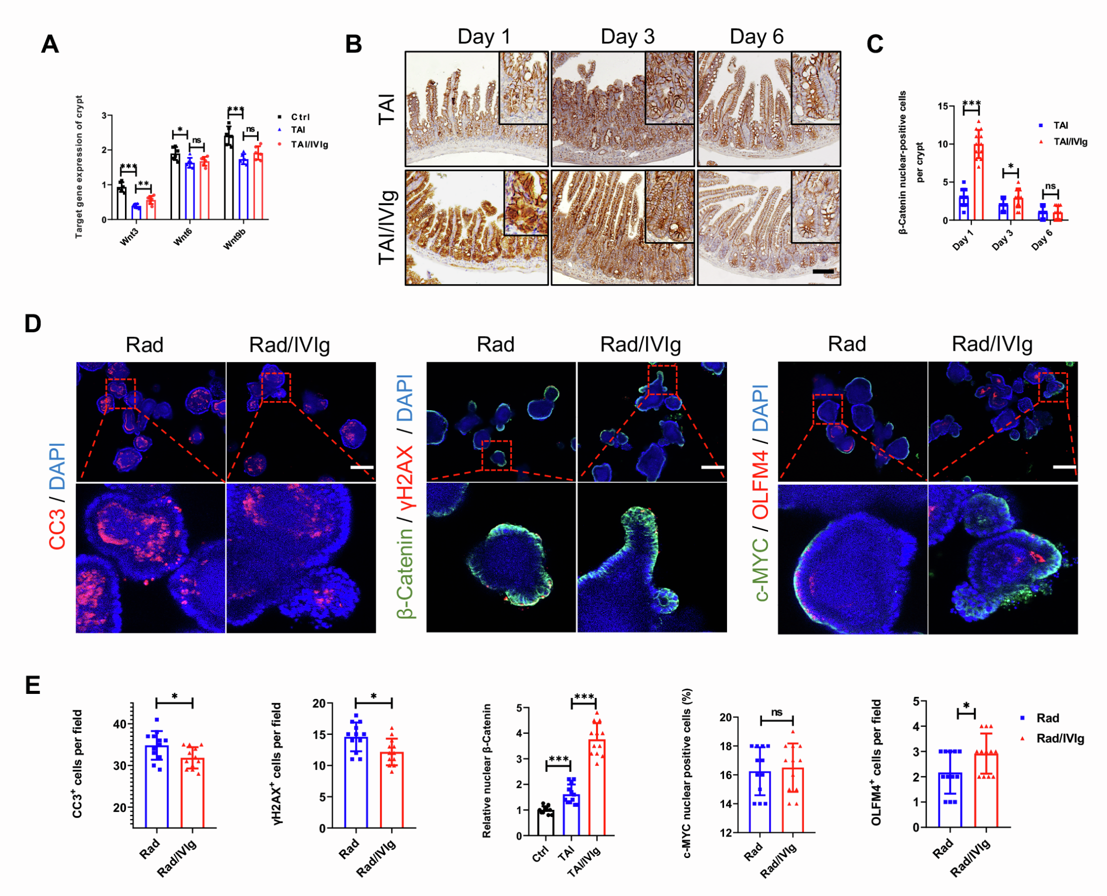

Figure S5. IVIg activates Wnt/β-Catenin signaling and alleviates radiation injury in intestinal crypts and organoids. (A) Changes in major *Wnt* subtypes in intestinal crypts of TAI mice after IVIg treatment. (B) Nuclear translocation of β-Catenin in mouse intestinal crypts shown by IHC. (C) Quantitative analysis of β-Catenin nuclear translocation. (D) Representative immunofluorescence images of Cleaved caspase-3, γ-H2AX, β-Catenin, OLFM4 and c-MYC and in enteroid. (E) Quantitative analysis of the markers shown in (D). * $p < 0.05$, ** $p < 0.01$, *** $p < 0.001$, ns = not significant.

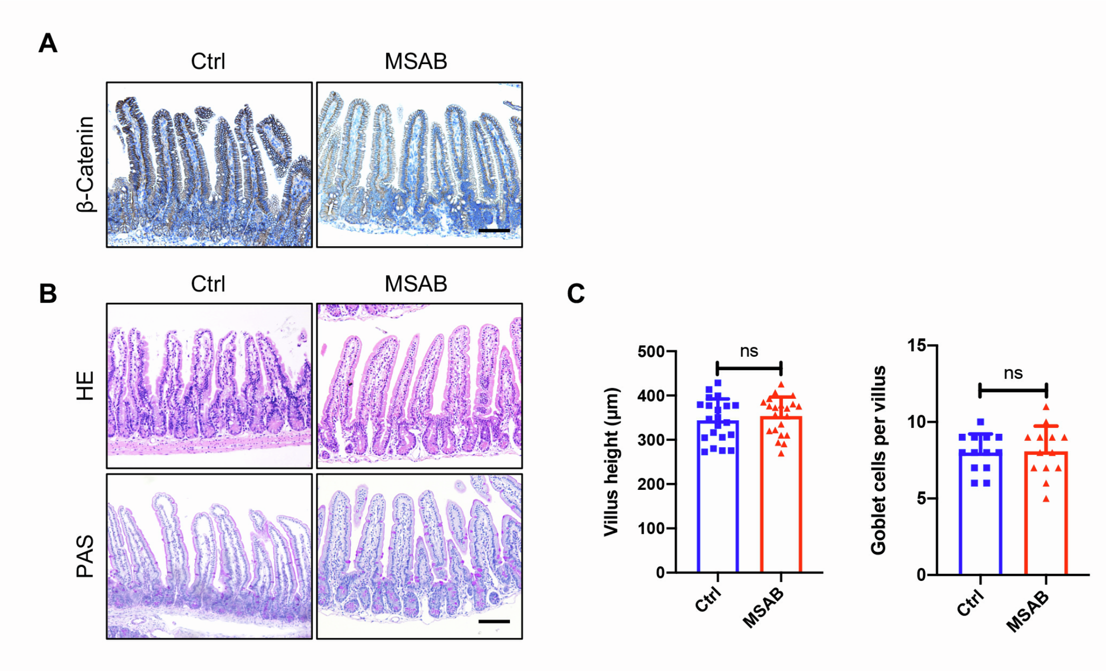

Figure S6. MSAB did not affect the intestine in short term. (A) Representative β-Catenin IHC-stained images of the small intestines (Bar = 100 μm). (B) Representative HE and PAS staining of the small intestines (Bar = 100 μm). (C) Statistical analysis of the villus heights and PAS^{+} cells, n > 10 villi. ns = not significant.

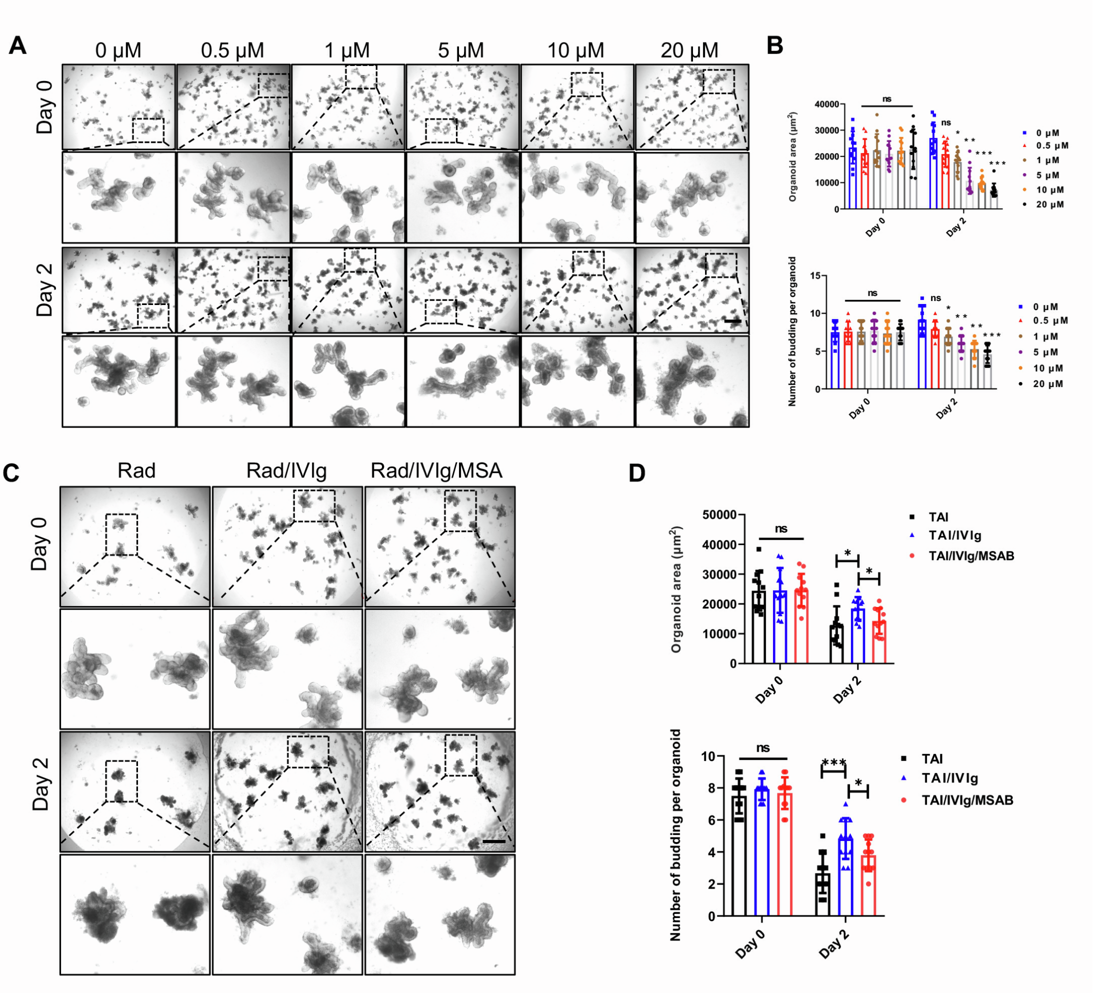

Figure S7. Blocking of the β-Catenin pathway eliminates the protective effect of IVIg on irradiation-damaged enteroids. (A) Microscopic images of mature enteroids after treatment with MSAB (Bar = 500 μm). (B) Quantitative analysis of enteroids area and budding number per enteroid, n > 10 crypts. (C) Microscopic images of enteroids after 6 Gy X-ray irradiation (scale bar = 500 μm). (D) Quantitative analysis of enteroids area and budding number per enteroid, n = 12 enteroids. * $p < 0.05$, ** $p < 0.01$, *** $p < 0.001$, ns = not significant.

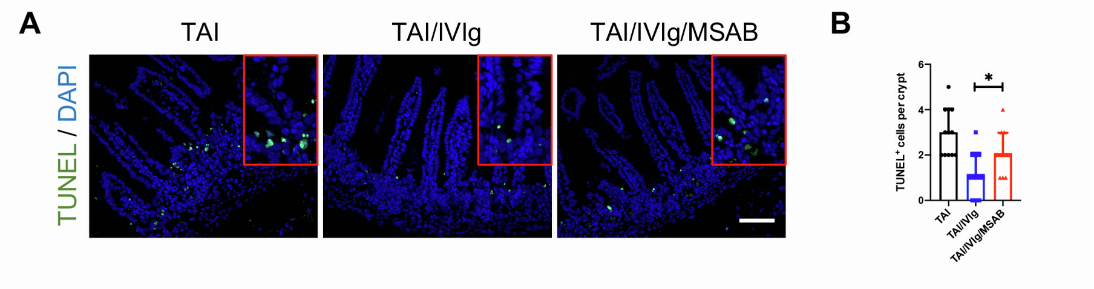

Figure S8. Blocking of the β-Catenin pathway eliminates the protective effect of IVIg against crypt apoptosis in TAI mice. (A) Representative TUNEL staining of small intestines (Bar = 100 μm). (B) Statistical analysis of TUNEL$^+$ cells, n > 10 crypts. * $p < 0.05$.
