# Supplementary material for: Intravenous immunoglobulin enhances intestinal stem cell regeneration to mitigate radiation-induced enteritis via promoting β-catenin nuclear translocation
Source: Stem Cell Reports. 2026 Jun 4;21(7):102934. doi: 10.1016/j.stemcr.2026.102934 (PMC13385420; doi:10.1016/j.stemcr.2026.102934)
Supplement: Document S2. Article plus supplemental information [file mmc2.pdf]

STEM CELL REPORTS
ISSCR
Article
OPEN ACCESS

Intravenous immunoglobulin enhances intestinal stem cell regeneration to mitigate radiation-induced enteritis via promoting β-catenin nuclear translocation

Jia He,[1,3,5] Tiancheng Chu,[1,5] Ping Fu,[1,5] Peng Jiang,[1] Li Ma,[1] Fengjuan Liu,[1] Xi Du,[1] Zhenni Xu,[2] Jun Xu,[4] Lu Cheng,[4] Changqing Li,[1,*] Dengqun Liu,[2,*] and Zongkui Wang[1,6,*]

[1]Institute of Blood Transfusion, Chinese Academy of Medical Sciences & Peking Union Medical College, Chengdu 610052, China
[2]Precision Radiation in Oncology Key Laboratory of Sichuan Province, Department of Experimental Research, Sichuan Cancer Hospital & Institute, Sichuan Provincial Engineering Research Center for Tumor Organoids and Clinical Transformation, Sichuan Clinical Research Center for Cancer, Sichuan Cancer Center, School of Medicine, University of Electronic Science and Technology of China, Chengdu 610041, China
[3]Basic Research Innovation Center for Acute Radiation Syndrome, Lab of Radiation Biology, Department of Blood Transfusion, Laboratory Medicine Center, The Second Affiliated Hospital of Army Medical University, Chongqing 400037, China
[4]Shanghai RAAS Blood products Co., Ltd., Shanghai 201401, China
[5]These authors contributed equally
[6]Lead contact
*Correspondence: lcq@ibt.pumc.edu.cn (C.L.), dengqunliu@uestc.edu.cn (D.L.), zongkui.wang@ibt.pumc.edu.cn (Z.W.)
https://doi.org/10.1016/j.stemcr.2026.102934

SUMMARY

Radiation-induced enteritis (RIE), a common adverse effect after radiotherapy for pelvic, abdominal, and retroperitoneal tumors, has no effective treatment. Our previous study showed that intravenous immunoglobulin (IVIg) ameliorated radiation toxicity. Here, we investigated the role of IVIg in enhancing intestinal stem cell (ISC) regeneration to mitigate RIE and its underlying mechanism. In a mouse RIE model, IVIg improved tissue repair, reduced intestinal epithelial apoptosis and pyroptosis, decreased inflammatory factors, enhanced antioxidant capacity, and alleviated DNA damage. Notably, IVIg promoted the proliferation of OLFM4^{+} and LGR5^{+} ISCs in crypts and mitigated radiation-induced enteroid damage *in vitro*. Lineage tracing revealed that IVIg enhanced LGR5^{+} ISCs and their daughter cell survival. Mechanistically, IVIg promoted β-catenin nuclear translocation, and the β-catenin inhibitor MSAB diminished IVIg's radioprotective effects. Collectively, IVIg combats RIE by enhancing ISC regeneration via β-catenin nuclear translocation, highlighting its potential as an RIE therapeutic candidate.

INTRODUCTION

Ionizing radiation exposures, such as those due to nuclear accidents, nuclear explosions, and radiotherapy, can cause serious damage to health. The extent of radiation-induced damage depends on the exposure dose, beam type, and radiosensitivity of the target organ (Gandle et al., 2020; Toullec et al., 2018). The gastrointestinal tract, especially the intestinal epithelium, is highly sensitive to radiation exposure, and humans who receive accidental exposure usually suffer from radiation-induced enteritis (RIE) (Bensemmane et al., 2021; Kwak et al., 2021). Radiotherapy in patients with pelvic, abdominal, and retroperitoneal tumors frequently results in either acute or chronic RIE, causing damage to the small or large intestine to different extents (Bhutta et al., 2023). It has been reported that approximately 20% of patients undergoing radiotherapy exhibit clinical symptoms of RIE, including abdominal pain, diarrhea, and malabsorption, which seriously limit the radiotherapy course (Loge et al., 2020). Although numerous studies have explored radioprotective agents, such as antioxidants, growth factors, and small chemical molecules, specific and effective treatments for RIE are still lacking, and physicians can merely alleviate the clinical symptoms of RIE (Fu et al., 2021; Rao et al., 2021; Wang et al., 2022; Xiao et al., 2020). Thus, developing new drugs for RIE treatment is urgently needed.

The pathophysiology of RIE has been extensively studied over the past decades. However, the underlying mechanism is complex and remains elusive (Fan et al., 2022). The intestinal epithelium with a single-layer structure undergoes vigorous renewal and is completely renewed within 3–5 days, which is attributed to the proliferation and differentiation of intestinal stem cells (ISCs) (Clevers, 2013; Gregorieff et al., 2015; Zhu et al., 2022). Wnt/β-catenin signaling is pivotal to both homeostatic maintenance and regeneration of ISCs, and it has been found that the Wnt protein can bind to ISC surface receptors, which leads to the nuclear translocation of β-catenin (de Lau et al., 2014; Yan et al., 2017). Through this pathway, ISCs can support the rapid self-renewal and regeneration of the intestinal epithelium. It has been shown that after intestinal irradiation, the downregulation of β-catenin signaling by damaging ISC regulatory factors in cryptal and mesenchymal cells can lead to impaired regeneration of the intestinal epithelium (Jun et al., 2016; Yang et al., 2021). Thus, activating Wnt/β-catenin signaling in the

© 2026 The Authors.
Published by Elsevier Inc. on behalf of International Society for Stem Cell Research.
This is an open access article under the CC BY license (http://creativecommons.org/licenses/by/4.0/).

injured intestine may stimulate the proliferation and differentiation of ISCs, which ultimately promote the repair and regeneration of the intestinal epithelium (Lee et al., 2018; Lindemans et al., 2015).

Intravenous immunoglobulin (IVIg), which is a therapeutic preparation of IgG derived from pooled plasma from thousands of healthy blood donors, contains a broad spectrum of various antibodies and exhibits the dual function of immune replacement and immune regulation. The main component of IVIg is IgG, which can recognize and bind to diverse pathogen antigens, activate the complement system, promote the macrophage response, and neutralize viruses and toxins (Howard et al., 2017; Willison et al., 2016). As approved by the US FDA, IVIg is used as a first-line treatment for a variety of autoimmune and inflammatory diseases. IVIg has been widely used clinically for the treatment of Guillain-Barre syndrome, myasthenia gravis, multifocal motor neuropathy, immune thrombocytopenia, and Kawasaki disease, and it is also used in selected cases of multiple sclerosis (Bussel et al., 2021; Ganigara et al., 2021; Gibbons and Klein, 2021; Howard et al., 2017; Willison et al., 2016; Zuercher et al., 2016). Furthermore, IVIg may be a potential therapeutic for refractory inflammatory bowel disease, as numerous studies have demonstrated its efficacy in treating dextran sulfate sodium-induced colitis in mouse models (Charlet et al., 2019; Kozicky et al., 2019; Levine et al., 1992). However, the effects of IVIg on radiation-induced intestinal injuries remain largely unexplored. Our previous study showed that IVIg alleviated radiation toxicity, including hematopoietic and gastrointestinal tract injuries, by regulating the gut microbiota (Wang et al., 2023). Moreover, we demonstrated that IVIg protected the integrity of the intestinal epithelial barrier and inhibited ferroptosis induced by radiation exposure (He et al., 2024). Nevertheless, the effect of IVIg on ISCs afflicted with radiation-induced intestinal injury in mice and the underlying mechanism of action are still unclear.

In the present study, we found that IVIg augmented the regeneration of ISCs and promoted recovery of the intestinal epithelium in RIE mice. The results showed that IVIg alleviated RIE by reducing apoptosis and pyroptosis in intestinal epithelial cells, promoting crypt regeneration, and alleviating DNA damage. In addition, IVIg rescued $OLFM4^+$ and $LGR5^+$ ISCs from radiation toxicity. Finally, the classical nuclear translocation of β-catenin played a pivotal role in IVIg-mediated intestinal regeneration after radiation-induced injury. To our knowledge, this study is the first on the regulation of ISC function after RIE by IVIg, and it suggests a novel and effective medical countermeasure to mitigate RIE in clinical practice.

RESULTS

IVIg mitigates radiation-induced intestinal toxicity in mice

To investigate the effect of IVIg on RIE, we first established a mouse model of 12 Gy single-dose total abdominal irradiation (TAI). 250, 500, and 1,000 mg/kg doses of IVIg were defined as IVIg-L, IVIg-M, and IVIg-H, respectively. We found that a moderate dose of 500 mg/kg was sufficient to preserve body weight and intestinal length in irradiated mice (Figures S1A and S1B). As shown in Figure 1A, C57BL/6J mice received an intravenous injection of IVIg at 500 mg/kg within 1 h of 12 Gy TAI, followed by repeated injection every three days. Compared with the control group, the TAI group exhibited a significant reduction in small intestine length, whereas IVIg treatment markedly increased the small intestine length (Figures 1B and 1C). The regenerative effects of IVIg on the intestine were further examined by microscopic analysis of the villus-crypt axis. The IVIg-treated mice showed improved crypt architecture and restored crypt depth compared with the TAI group on days 3 and 6, which indicated mitigation of radiation-induced intestinal damage (Figures 1D and 1E). Both prophylactic (1 h before irradiation) and therapeutic (1 h after irradiation) intravenous administration of IVIg conferred consistent protective effects (Figures S1C and S1D). Furthermore, IVIg administration significantly decreased the number of cleaved caspase-3^+ (CC3) crypt cells on day 1 post-TAI, demonstrating that IVIg prominently suppressed cell apoptosis in the crypts (Figures 1F and 1G). On day 3, the TAI group showed a substantial increase in gasdermin D-positive ($GSDMD^+$) cells in the whole intestinal epithelium, whereas the shear band of N-terminal fragment of gasdermin D (GSDMD-N) was markedly increased in intestinal crypts. Remarkably, IVIg administration significantly mitigated pyroptosis in the intestinal epithelium (Figures 1H and 1I). Collectively, these results underscored the protective efficacy of IVIg in mitigating intestinal toxicity caused by TAI.

IVIg mitigates the inflammatory response, oxidative stress, and DNA damage induced by TAI

Radiation injury usually triggers systemic inflammatory reactions and oxidative stress. On day 2 after TAI, it was found that the IVIg-treated mice had longer small intestines than the control mice, and inflammatory exudate was clearly observed in the intestinal lumen of the TAI mice but not in the TAI/IVIg group (Figure 2A). This finding implied that IVIg might be capable of alleviating inflammation and oxidative stress after TAI. Therefore, we subsequently examined the levels of inflammatory factors and antioxidants using ELISA. The results

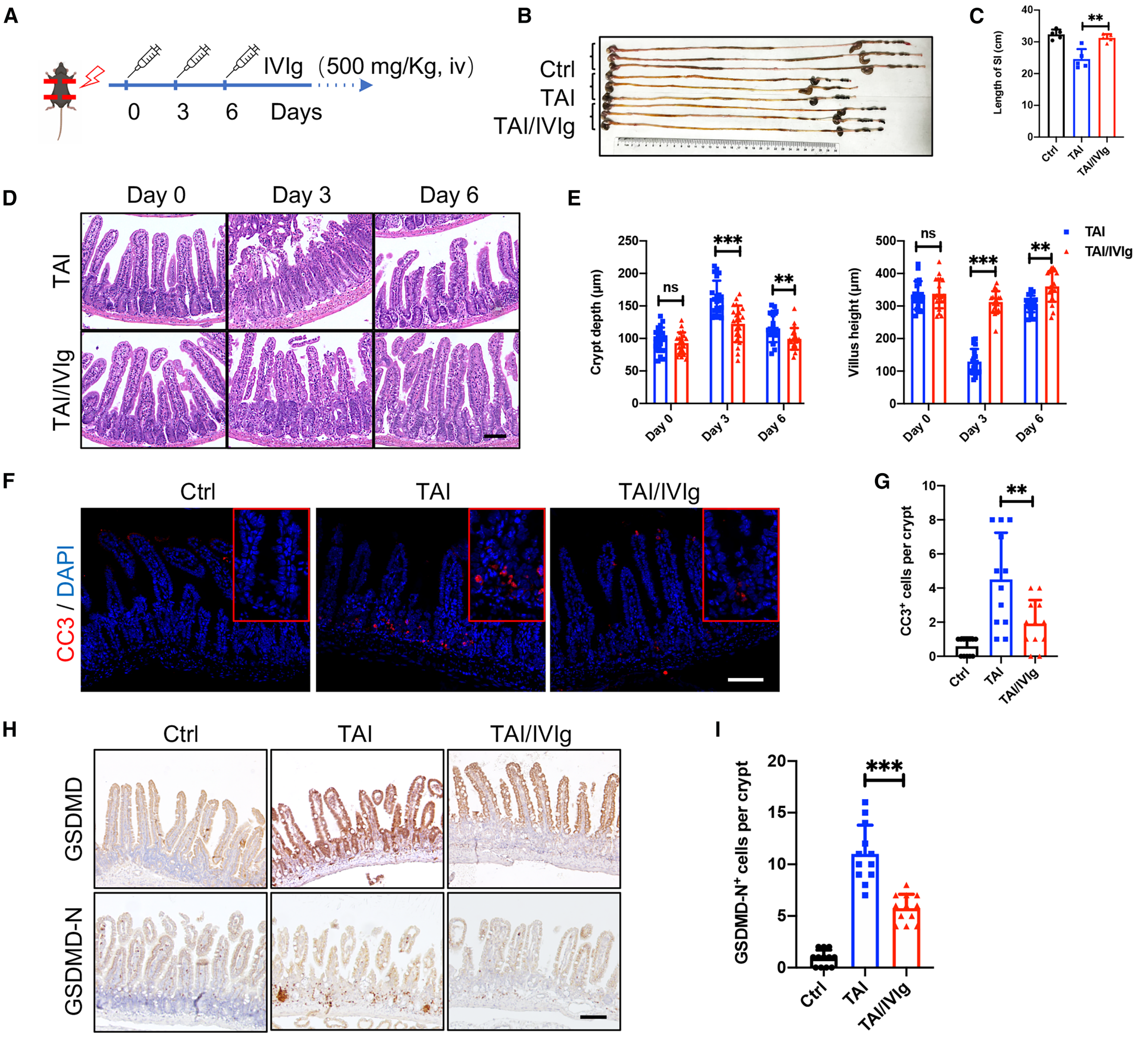

Figure 1. IVIg mitigates radiation-induced intestinal toxicity in mice
(A) 12 Gy TAI irradiation and administration regimen.
(B) Comparison of intestinal gross morphology on day 3 after TAI.
(C) Statistical analysis of small intestinal length on day 3 after TAI, $n = 5$.
(D) Representative images of small intestinal H&E staining on days 0, 3, and 6 after TAI (scale bars, 100 μm).
(E) Statistical analysis of crypt depth and villus height in mouse small intestine; $n > 15$ crypts/villi per group.
(F) Representative images of CC3 staining in small intestinal crypts on day 1 after 12 Gy TAI (scale bars, 100 μm).
(G) Comparison of the number of CC3-positive cells in mouse crypts; $n = 12$ crypts.
(H) Immunohistochemical staining of GSDMD and GSDMD-N in mouse small intestine on day 3 after TAI (scale bars, 100 μm).
(I) Quantitative comparison of GSDMD-N-positive cells; $n = 12$ crypts. **, $p < 0.01$; ***, $p < 0.001$; ns, not significant.

demonstrated that IVIg significantly decreased the elevated levels of interleukin 1β (IL-1β), IL-6, and IL-17 and interferon γ (IFN-γ) in the small intestines of the TAI mice (Figure 2B). Compared with those in the untreated group, the superoxide dismutase (SOD)and glutathione (GSH) levels were significantly decreased in the TAI group and significantly ameliorated by IVIg treatment (Figure 2C), suggesting that IVIg could attenuate RIE by reducing oxidative stress. Additionally, DNA damage was analyzed in the TAI and TAI/IVIg groups. Strong

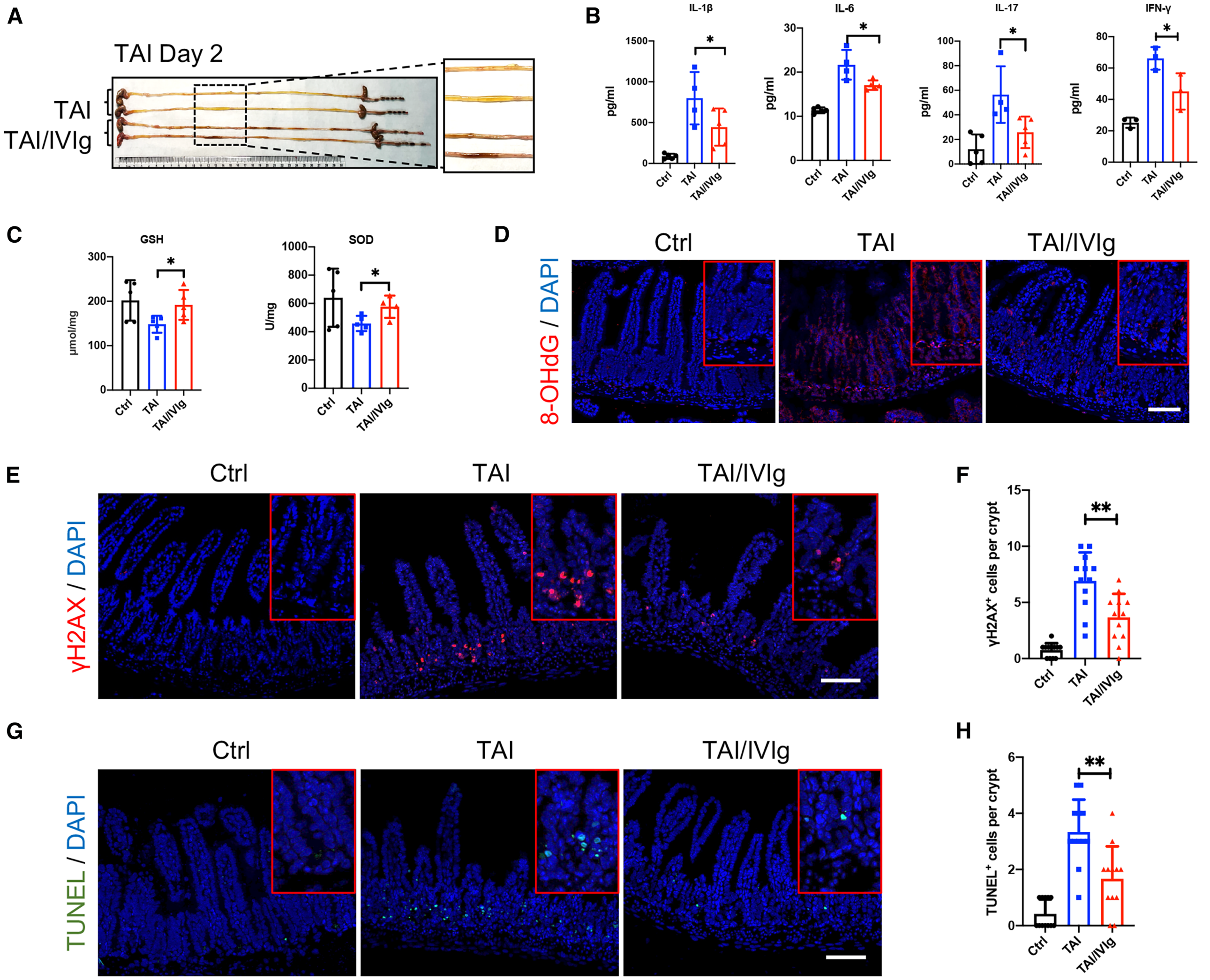

Figure 2. IVIg attenuated intestinal inflammatory oxidative stress and reduced DNA damage and apoptosis after TAI treatment
(A) Intestinal bright-field images on day 2 after TAI, $n = 5$.
(B) ELISA of IL-1β, IL-6, IL-17, and IFN-γ levels in small intestinal tissues on day 2 after TAI ($n = 4$ or 5).
(C) Intestinal oxidative stress levels on day 2 after TAI.
(D) 8-OHdG immunofluorescence staining on day 2 after TAI (scale bars, 100 μm).
(E) Immunofluorescence staining of γH2AX-positive cells in small intestinal crypts on day 1 after TAI (scale bars, 100 μm).
(F) Quantification of γH2AX-positive cells in mouse small intestinal crypts on day 1 after 12 Gy TAI; $n > 10$ crypts.
(G) Representative images of TUNEL-positive cells on day 1 after TAI (scale bars, 100 μm).
(H) Quantification of TUNEL-positive cells in mouse crypts; $n > 10$ crypts per group.
*, $p < 0.05$; **, $p < 0.01$.

8-hydroxy-2′-deoxyguanosine (8-OHdG) staining was observed at 24 h in the TAI group, whereas staining was significantly reduced in the TAI/IVIg intestine (Figure 2D). Moreover, $\gamma H2AX^+$ signals were obviously increased in the crypts at 24 h after TAI, indicating severe DNA damage, but there was a significant decrease in γH2AX signals in the crypts of mice receiving IVIg treatment (Figures 2E and 2F). A significant increase in the number of $TUNEL^+$ cells was observed in the crypts at 24 h after TAI, but the number decreased in the IVIg-treated group, indicating that IVIg mitigated radiation-induced apoptosis in the crypts (Figures 2G and 2H).

Collectively, these data demonstrate that IVIg reduces radiation-induced inflammation, oxidative stress, DNA damage, and cell apoptosis, and, thus, IVIg treatment improves the microenvironment for ISC survival, which is necessary

for the rapid regeneration of the intestinal epithelium after RIE.

IVIg promotes proliferation and maintains epithelial lineages in the small intestine after radiation-induced injury

Ki67 immunohistochemistry (IHC) staining showed that $Ki67^+$ crypt cells decreased on day 1 after TAI and recovered gradually by days 3 and 6. IVIg treatment markedly increased $Ki67^+$ cells, especially proliferative ISCs, at the crypt base (red arrows, Figure 3A). Statistical results are consistent with this trend (Figure 3B). Intestinal tissues were collected 90 min after BrdU injection for BrdU immunofluorescence (IF) staining. Clear $BrdU^+$ crypt cells were observed in the control group, whereas their number was markedly decreased in the TAI group on day 3 after TAI. IVIg treatment significantly increased the number of $BrdU^+$ cells in the crypts, suggesting that IVIg promotes DNA synthesis and cell proliferation following TAI-induced intestinal injury (Figures 3C and 3D). IVIg administration also increased the number of goblet cells (PAS^+) in the villi of the TAI/IVIg mice compared with that in the TAI group on day 3 after irradiation (Figures 3E and 3F). Similarly, the mice in the TAI/IVIg group showed significant increases in the numbers of both Paneth cells ($LYSOZYME^+$) and absorptive epithelial cells ($FABP1^+$) compared with those in the TAI group (Figure 3G). The number of CgA^+ (chromogranin A) cells was significantly reduced 3 days after TAI. IVIg treatment contributed to a significant recovery in the number of CgA^+ cells (Figure S2). Transcriptome analysis indicated that IVIg upregulated the expression of multiple genes associated with cell proliferation in RIE mice, thereby exerting a protective effect against RIE (Figure 3H). Collectively, these findings demonstrate that IVIg enhances the proliferative activity of crypt epithelial cells and preserves intestinal epithelial structure following TAI-induced intestinal damage.

IVIg increases the survival of enteroids after radiation-induced toxicity

To investigate the *in vitro* effect of IVIg on RIE, an enteroid model was established (Figure S3A). Our data revealed that a minimum concentration of 2.5 mg/mL IVIg promoted the formation of crypt-like buds in the enteroids (Figures S3B and S3C). The enteroids were exposed to a single 6 Gy X-ray irradiation, and the experimental group was treated with IVIg immediately after irradiation. IVIg treatment significantly increased the survival rate of enteroids on day 3 and promoted their regeneration by day 6 (Figures 4A and 4B). As shown by H&E staining in Figure 4C, the structure of enteroids in the IVIg-treated group was more intact and comparable to that in the control group. In contrast, isolated $F(ab')_2$ fragments of IVIg and IgG1 did not mimic the protective effect of intact IVIg (Figure S4). These findings suggest that the protective effect of IVIg in irradiated intestinal organoids is Fc dependent and requires IVIg-specific properties. Moreover, an increased presence of $Ki67^+$ proliferative epithelial cells in the IVIg-treated enteroids was found by confocal IF, suggesting that IVIg was capable of promoting cell proliferation of radiation-damaged enteroids (Figure 4D). The results of IF staining showed that γH2AX and 8-OHdG fluorescence was less obvious in the IVIg-treated enteroids, suggesting that IVIg was able to protect the DNA of radiation-damaged intestinal cells in the enteroid model (Figure 4E). In addition, IVIg-treated enteroids displayed fewer apoptotic cells than the irradiation group (Figure 4F). Consequently, these results support that IVIg can directly impact the cryptal epithelium and potentially enhance the survival of ISCs, which serve as the progenitors of daughter epithelial cells in enteroids.

IVIg promotes the survival and differentiation of ISCs after RIE

OLFM4 IHC staining showed that the number of $OLFM4^+$ ISCs was markedly reduced on days 1 and 3 after 12 Gy TAI. Administration of IVIg significantly increased the number of $OLFM4^+$ ISCs, and the two groups exhibited comparable numbers on day 6 (Figure 5A). Statistical results are consistent with this trend (Figure 5B). Furthermore, the crypts were isolated on day 3 after irradiation, and western blot was used to detect OLFM4 protein expression. The results showed higher OLFM4 protein levels in the IVIg-treated group (Figure 5C). These findings indicated that IVIg attenuates the reduction in $OLFM4^+$ ISCs caused by radiation injury and accelerates their recovery. Subsequently, LGR5 IHC staining revealed that the number of $LGR5^+$ ISCs in IVIg-treated mice was significantly higher than that in the control group on day 3 after irradiation (Figures 5D and 5E). To further verify the effect of IVIg on ISCs, *Lgr*-EGFP-IRES-CreERT2/Rosa26-tdTomato mice were used to label and trace $LGR5^+$ ISCs. Tamoxifen was injected intraperitoneally within 1 h of irradiation, and the mice were sacrificed 24 h later. In the TAI group, $LGR5^+$ ISCs and $tdTomato^+$ progeny were almost completely lost, whereas IVIg-treated mice retained viable $EGFP^+$ $LGR5^+$ ISCs that generated new $tdTomato^+$ epithelial daughter cells (Figure 5F). Quantification confirmed more *Lgr*-$EGFP^+$ ISCs and $tdTomato^+$ epithelial cells in the crypts of the IVIg group (Figure 5G). Crypts isolated from these mice were cultured as enteroids and labeled with 4-hydroxytamoxifen before 6 Gy irradiation. *In vitro*, irradiation markedly reduced $EGFP^+$ $LGR5^+$ ISCs and $tdTomato^+$ progeny, while IVIg treatment preserved both populations (Figure 5H). These results support that IVIg enhances ISC function and ameliorates TAI-induced intestinal injury.

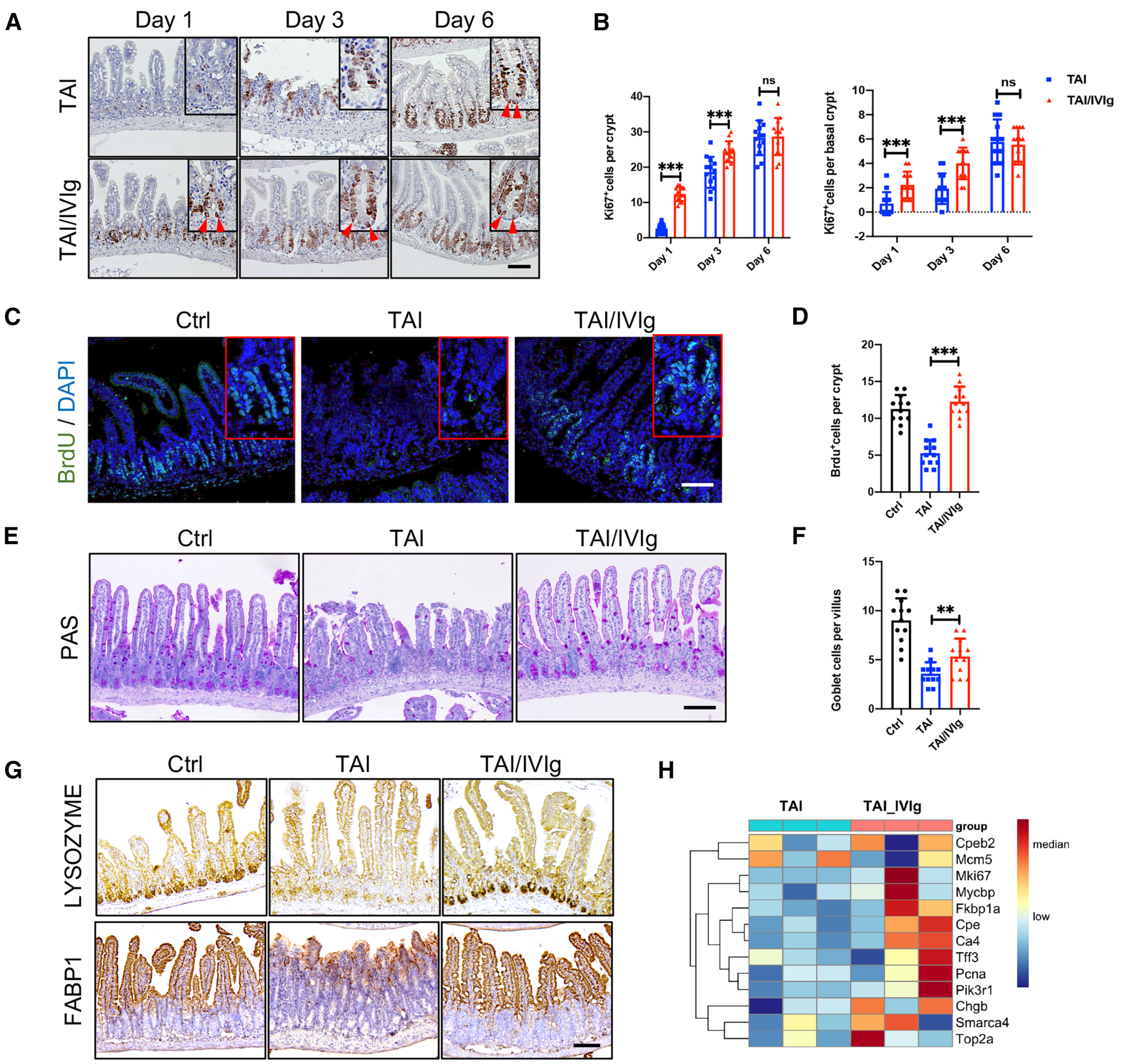

Figure 3. IVIg increased the proliferation capability and differentiation toward goblet cells, Paneth cells, and absorptive epithelial cells in the small intestines after TAI

(A) Representative images of Ki67 immunohistochemical staining of regenerating crypts in the small intestine on days 1, 3, and 6 after 12 Gy TAI (scale bars, 100 μm).
(B) Quantitative analysis of Ki67-positive cells in the whole crypt and crypt base; $n = 12$ crypts.
(C) Representative images of BrdU immunofluorescence staining of small intestinal crypts on day 3 after 12 Gy TAI (scale bars, 100 μm).
(D) Quantitative analysis of BrdU-positive cells in small intestinal crypts; $n = 12$ crypts.
(E) PAS staining of intestinal epithelial goblet cells on day 3 after TAI (scale bars, 100 μm).
(F) Quantitative analysis of PAS-positive cells in the intestine on day 3 after TAI; $n = 12$ crypts.
(G) Representative images of LYSOZYME and FABP1 immunohistochemical staining of the mouse small intestine on day 3 after TAI; $n = 6$.
(H) Heatmap of small intestinal transcriptome sequencing showing the expression of genes related to cell proliferation and differentiation; $n = 3$ mice. **, $p < 0.01$; ***, $p < 0.001$; ns, not significant.

IVIg promotes the nuclear translocation of β-catenin to augment the survival of ISCs and epithelial regeneration after TAI-induced injury

Analysis of intestinal transcriptome sequencing data identified three significantly different gene clusters (cluster 2, cluster 6, and cluster 7) for further in-depth analysis (Figure 6A). Differentially expressed genes (DEGs) between the TAI/IVIg and TAI groups were analyzed and visualized using volcano plots (Figure 6B). The target gene clusters were intersected with DEGs in the TAI/IVIg group versus the TAI group, and 161 genes were screened by Venn diagram (Figure 6C). Further Gene Ontology (GO) and Kyoto Encyclopedia of

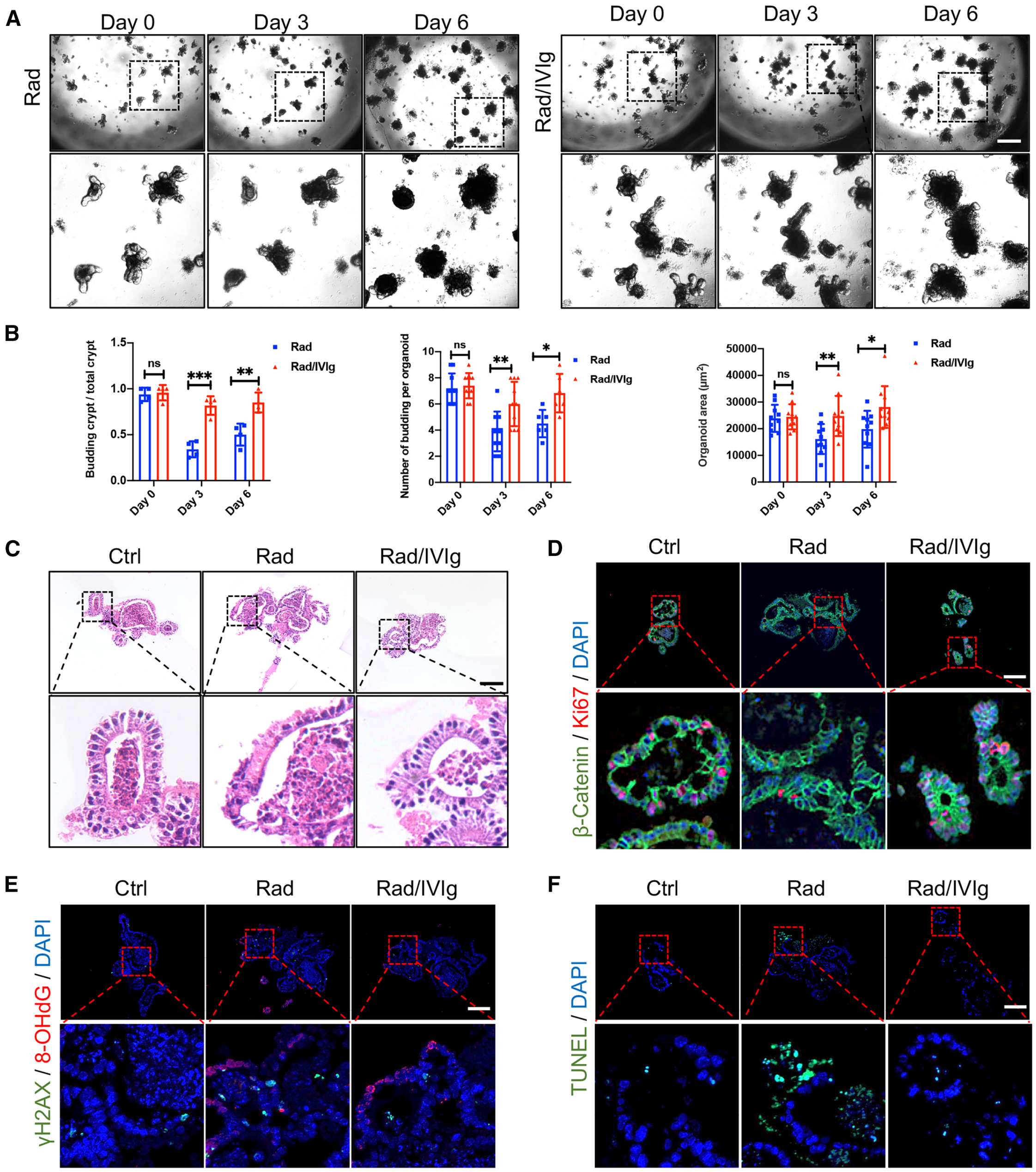

Figure 4. IVIg alleviated radiation-induced toxicity and promoted cell proliferation in enteroids

(A) Microscopic images of enteroids on days 0, 3, and 6 after 6 Gy X-ray irradiation (scale bars, 500 μm).

(B) Quantitative analysis of enteroids on days 0, 3, and 6 after 6 Gy irradiation, including the percentage of budding enteroids, enteroid area, and budding number per enteroid. Three independent biological replicates (from 3 mice) were analyzed, and 12 enteroids were counted.

(C) Representative images of H&E staining of enteroids in each group on day 2 after 6 Gy irradiation (scale bars, 100 μm).

(D) Immunofluorescence staining of Ki67 (red) and β-catenin (green) in enteroids on day 2 after 6 Gy irradiation (scale bars, 100 μm). Data are representative of three independent experiments.

(E) Representative images of γH2AX (green) and 8-OHdG (red) immunofluorescence staining of enteroids after 6 Gy irradiation.

(F) Representative images of TUNEL-positive cells on day 2 after 6 Gy irradiation (scale bars, 100 μm). *, $p < 0.05$; **, $p < 0.01$; ***, $p < 0.001$; ns, not significant.

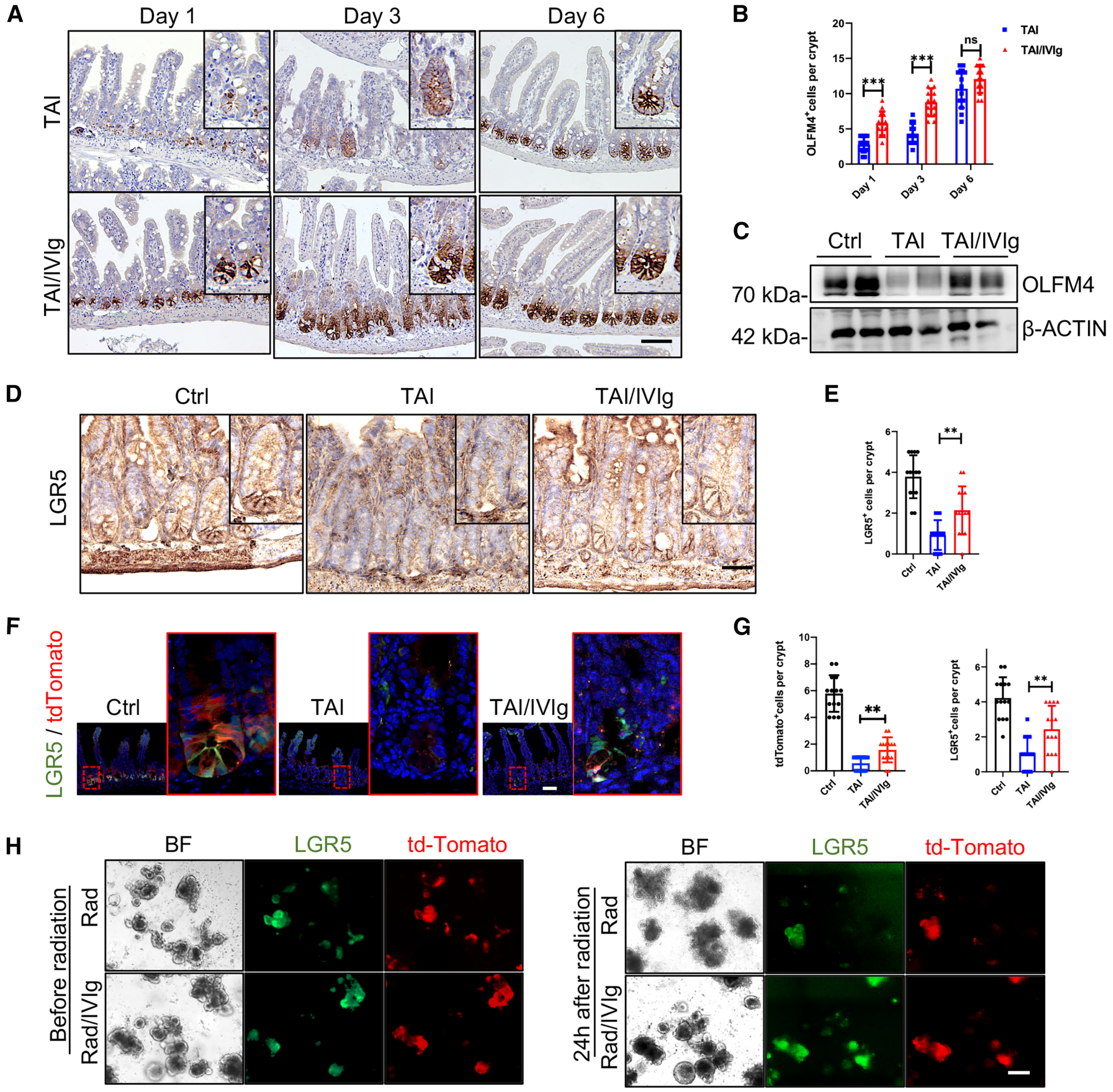

Figure 5. IVIg promoted the survival of OLFM4^{+} and LGR5^{+} ISCs after 12 Gy TAI injury

(A) Representative images of OLFM4 immunohistochemical staining of the mouse small intestine on days 1, 3, and 6 after 12 Gy irradiation (scale bars, 100 μm).

(B) Quantitative analysis of OLFM4-positive cells; n = 12 crypts per group.

(C) Western blotting detection of OLFM4 protein in the small intestine.

(D) Representative images of LGR5^{+} ISCs in crypts on day 3 after 12 Gy TAI (scale bars, 200 μm).

(E) Quantitative analysis of LGR5^{+} crypt cells in each group; n = 12 crypts.

(F) Lineage tracing images of *Lgr*-EGFP-positive ISCs (green) and their tdTomato-positive progeny (red) at 24 h after 12 Gy TAI (scale bars, 100 μm).

(G) Quantitative analysis of LGR5^{+} ISCs and their progeny; n = 12 crypts.

(H) *In vitro* lineage tracing images of LGR5^{+} ISCs (green) and their tdTomato-positive progeny (red) in enteroids at 24 h after 6 Gy irradiation (scale bars, 100 μm). Six mice were used per group in animal experiments.

*, $p < 0.05$; **, $p < 0.01$; ***, $p < 0.001$; ns, not significant.

Genes and Genomes (KEGG) enrichment analyses of these genes revealed that the Wnt/β-catenin pathway mediates the protective effect of IVIg on the intestine in TAI-treated mice (Figures 6D and 6E). Reverse-transcription polymerase chain reaction (RT-PCR) was performed to detect the expression of the three major Wnt subtypes in the intestine, showing that IVIg mainly regulated the *Wnt3a* subtype (Gregorieff et al., 2005) (Figure S5A).

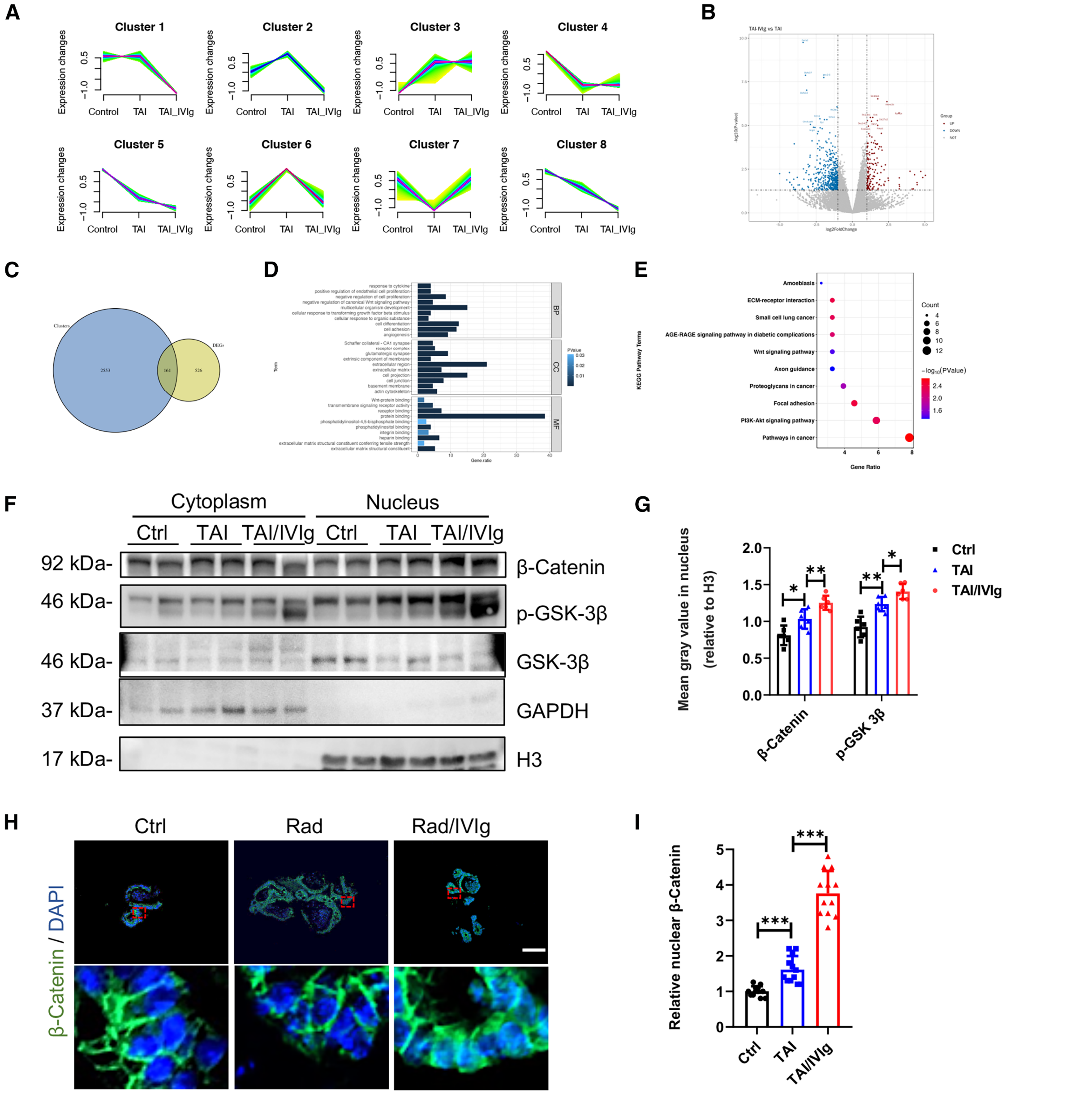

Figure 6. IVIg activated the β-catenin pathway after TAI to augment the survival of ISCs

(A) Cluster analysis of gene transcriptome sequencing.

(B) Volcano plot of differentially expressed genes; $n = 3$ mice.

(C) Venn diagram of differentially expressed genes in clusters 2, 6, and 7.

(D) GO functional enrichment analysis of the overlapping genes in the Venn diagram.

(E) KEGG pathway analysis of the overlapping genes in the Venn diagram.

(F) Western blotting results of β-catenin, GSK3β, and *p*-GSK3β in the cytoplasm and nucleus of mouse intestinal crypts on day 2 after 12 Gy irradiation.

(G) Quantitative analysis of western blotting results.

(H) Representative images of β-catenin (green) immunofluorescence staining in mouse enteroids on day 2 after 6 Gy irradiation (scale bars, 100 μm).

(I) Quantitative analysis of nuclear β-catenin expression in enteroids after 6 Gy irradiation.

*, $p < 0.05$; **, $p < 0.01$; ***, $p < 0.001$.

IHC results showed that β-catenin was barely detectable in the nuclei of intestinal tissues on day 1 after TAI-induced intestinal injury, but its expression gradually increased at day 3 and recovered to normal levels by day 6. Compared with the TAI group, prominent expression of β-catenin was observed in the nuclei of small intestinal crypts in the IVIg-treated group at day 1 post-TAI injury (Figures S5B and S5C). Furthermore, western blot analysis was performed to detect the expression of β-catenin pathway-related proteins in the nucleus and cytoplasm of the small intestinal crypts at 24 h post-injury. The results revealed that the nuclear expression levels of β-catenin and *p*-GSK-3β in intestinal crypts were significantly elevated in the TAI/IVIg group compared with those in the TAI group (Figures 6F and 6G), suggesting that IVIg promotes the activation of the β-catenin pathway. *In vitro* experiments showed that 12 h after 6 Gy irradiation, the IVIg group exhibited weaker positive signals for γH2AX and CC3 in the intestinal enteroids compared with the control group, while nuclear β-catenin localization and the expression of its downstream target c-Myc showed no significant increase, and OLFM4 expression was elevated (Figures S5D–S5E).

At 48 h post-irradiation, only faint nuclear β-catenin staining was observed, which was markedly enhanced by IVIg treatment (Figures 6H and 6I). These results indicated that IVIg promotes the repair of ISCs through the β-catenin pathway, with the key mechanism being the promotion of β-catenin nuclear translocation. The absence of β-catenin activation in the acute phase suggests that IVIg may also act directly via a Wnt-independent cytoprotective mechanism.

Blocking β-catenin signaling abolishes the protective effect of IVIg on ISCs after TAI-induced intestinal injury

To inhibit β-catenin signaling, the mice were intraperitoneally injected with 15 mg/kg methyl 3-{[(4-methylphenyl)-sulfonyl]amino}benzoate (MSAB), a β-catenin inhibitor (Hwang et al., 2016), once daily for 2 consecutive days before TAI. The IHC staining results revealed a significant decrease in β-catenin expression in the intestines of the MSAB-treated mice compared with that in the control group, whereas H&E and PAS staining showed no observable histological changes in the mice after 2 days of pretreatment with MSAB (Figure S6). On day 3 following TAI and MSAB treatment, the mice were sacrificed. The gross images of the intestines showed that the protective effect of IVIg against TAI-induced intestinal damage in the mice was noticeably abolished because of the injection of MSAB (Figures 7A and 7B). H&E staining showed that the intestines from the MSAB-treated mice were worse than those from the TAI/IVIg mice (Figure 7C), with a notable decrease in the villus height observed in the MSAB group (Figure 7D).

In vitro experiments showed that after optimizing the concentration of MSAB to inhibit the growth of enteroids (Figures S7A and S7B), the protective effect of IVIg on irradiation-treated enteroids disappeared after the addition of MSAB (Figures S7C and S7D). The IF staining results indicated the presence of a higher number of $\gamma H2AX^{+}$ and $CC3^{+}$ cells in the TAI/IVIg/MSAB group than in the TAI/IVIg group (Figures 7E and 7F). In addition, the number of $TUNEL^{+}$ cells in the TAI/IVIg/MSAB group was higher than that in the TAI/IVIg group (Figure S8). As shown in Figures 7G and 7H, there were fewer $BrdU^{+}$ and $Ki67^{+}$ proliferating cells in the TAI/IVIg/MSAB group than in the TAI/IVIg group. Compared with that in the TAI/IVIg group, the number of $OLFM4^{+}$ stem cells was also significantly decreased in the TAI/IVIg/MSAB group (Figures 7I and 7J).

DISCUSSION

In both clinical practice and nuclear emergency preparedness, exploring potential drugs that can prevent crypt damage and promote epithelial repair and regeneration is critical to mitigating radiation damage (Leibowitz et al., 2021; Reynolds et al., 2014). However, finding suitable candidates that are devoid of significant side effects poses a challenge (Ren et al., 2016; Wang et al., 2020). In our previous study, IVIg was shown to exert anti-radiation effects by regulating the intestinal microbiota (Wang et al., 2023). As a widely used blood product with a favorable safety profile and wide clinical applications, IVIg has attracted our attention.

In this study, we found that IVIg administration following TAI modulated the inflammatory response in the small intestine of the mice. Additionally, IVIg treatment was shown to alleviate oxidative stress, reduce DNA damage in cryptal cells, and attenuate apoptosis and pyroptosis in cryptal epithelial cells, thereby mitigating TAI-induced intestinal damage (Figures S1 and S2). In addition, IVIg could promote cell proliferation in intestinal crypts and the differentiation of intestinal epithelial cells (Figure 3). Furthermore, we validated the reliability of the radio-protection effects of IVIg *in vitro* by using a mouse enteroid model (Figure 4), which suggested that IVIg could directly impact the intestinal epithelial cells. Notably, IVIg treatment enhanced cell proliferation during crypt regeneration in mice with RIE. In addition, IVIg treatment promoted the survival of $OLFM4^{+}$ and $LGR5^{+}$ ISCs (Figure 5). Finally, we found that IVIg enhanced the process of intestinal regeneration after RIE by promoting ISC-mediated crypt regeneration and repair by facilitating the nuclear translocation of β-catenin (Figures 6 and 7).

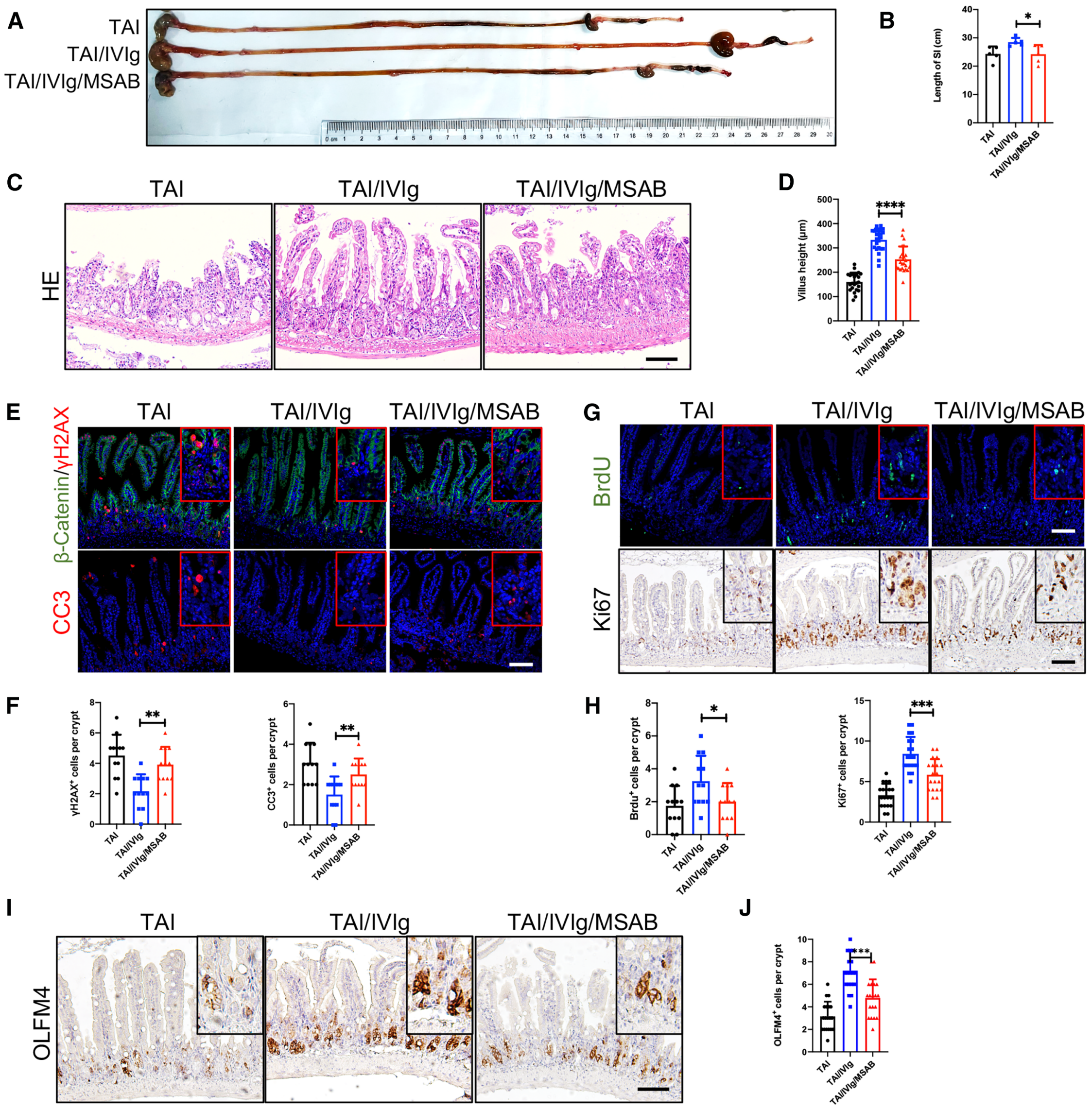

Figure 7. Blocking of the β-catenin pathway abolished the protective effects of IVIg on TAI-induced intestinal damage
(A) Representative images of mouse intestines on day 3 after TAI irradiation.
(B) Statistical analysis of small intestinal length; $n = 5$ mice.
(C) Representative images of small intestinal H&E staining (scale bars, 100 μm).
(D) Statistical analysis of small intestinal villus height; $n = 12$ crypts.
(E) Images of γH2AX/β-catenin staining and CC3 immunofluorescence in mouse intestines (scale bars, 100 μm).
(F) Quantitative analysis of γH2AX-positive and CC3-positive cells; $n = 12$ crypts.
(G) Representative images of BrdU-positive and Ki67-positive cells in the mouse small intestine (scale bars, 100 μm).
(H) Quantitative analysis of BrdU-positive and Ki67-positive cells; $n = 12$ crypts.
(I) Representative images of OLFM4-positive cells in the mouse small intestine (scale bars, 100 μm).
(J) Quantitative analysis of OLFM4-positive cells; $n = 12$ crypts.
*, $p < 0.05$; **, $p < 0.01$; ***, $p < 0.001$.

Rapid-cycling ISCs serve as the primary source for daily renewal of the intestinal epithelial cells. However, ISCs are fragile and susceptible to damage from a range of detrimental factors, such as ischemia, infection, and radiation exposure (Gregorieff et al., 2015). Numerous studies have increasingly demonstrated the significance of ISC survival following radiation exposure for the regeneration of epithelial cells and recovery of gastrointestinal mucosal

barrier function (Gong et al., 2016; Metcalfe et al., 2014; Saha et al., 2016). LGR5^{+} cryptal basal columnar cells are widely recognized as active ISCs that play a crucial role in the regeneration of the intestine after radiation exposure (Metcalfe et al., 2014). Additionally, OLFM4 can serve as a valuable marker for identifying active ISCs in both the human small intestine and colon, as it labels a broader range of positive cells than LGR5 (van der Flier et al., 2009). When RIE occurs, IVIg protects LGR5^{+} and OLFM4^{+} stem cells in the mice intestine, thereby promoting damage repair of the intestinal epithelium (Figures 5A–5E). Lineage tracing of the LGR5 intestinal trunk also indicated that IVIg had an effective protection against the differentiation of LGR5^{+} ISCs and their daughter cells (Figures 5F and 5G).

To uncover the potential signaling pathways through which IVIg acts on ISC proliferation and crypt growth after radiation-induced injury, we analyzed transcriptomic data for small intestinal tissues and found that the Wnt signaling pathway was significantly enriched (Figures 6A–6F). Previous studies have consistently demonstrated that the Wnt/β-Catenin pathway is closely related to stem cell control, disease development prognosis, and ISC repair after injury (He et al., 2004; Nusse and Clevers, 2017; Yan et al., 2017). Three days after irradiation, the activation of the small intestinal β-catenin pathway and nuclear translocation of the β-catenin protein are most prominent (Jang et al., 2022; Yang et al., 2021). Studies have reported that IVIg activates β-catenin and upregulates *Wnt5a* secretion in human dendritic cells, and the activation of β-catenin by IVIg requires intact IgG molecules and LRP5/6 co-receptors (Karnam et al., 2020).

In the present study, we found that IVIg administration accelerated the nuclear translocation of the β-catenin protein, causing it to occur much earlier (one day vs. three days after irradiation) both *in vivo* and *in vitro*. These results indicated that IVIg accelerated repair and regeneration of the small intestine in mice with TAI and rapidly initiated the repair program following radiation injury. This may provide an explanation for the heightened activation of the β-catenin signaling pathway in mice from the TAI/IVIg group on day 1 after radiation exposure, which subsequently significantly decreased by day 3, with protein levels similar to those of the TAI group (Figure 6). However, β-catenin is not activated during the acute injury phase, suggesting that IVIg may also exert a direct, Wnt-independent cytoprotective effect, whose early protective mechanism remains to be elucidated (Figures S5D and S5E). Furthermore, it was confirmed that the IVIg-mediated repair and regeneration in the small intestine were significantly attenuated when the β-catenin pathway was inhibited by MSAB, revealing that the β-catenin signaling pathway is important for the protective effects of IVIg against irradiation. In addition, we demonstrated that the protective effect of IVIg on irradiated intestinal epithelium is dependent on the Fc fragment and requires IVIg-specific properties in the intestinal organoid model (Figure S6).

While this study made several intriguing discoveries, it also has certain limitations. First, IVIg consists of thousands of antibodies, which pose challenges to the identification of specific antibodies responsible for its protective effects on ISCs. Meanwhile, the complexity of IVIg limits our ability to completely block its biological effects using chemical inhibitors. Consequently, we employed only MSAB, an inhibitor of β-catenin signaling, to block the downstream pathway regulated by IVIg. Moreover, IVIg products may vary slightly depending on plasma donor populations and regional sources used for IVIg manufacturing. Thus, there may be more or less differences in the experimental effects.

RESOURCE AVAILABILITY

Lead contact

Requests for further information, resources, and reagents should be directed to and will be fulfilled by the lead contact, Zongkui Wang (zongkui.wang@ibt.pumc.edu.cn).

Materials availability

This study did not generate new unique reagents.

Data and code availability

The RNA-seq data have been deposited in the NCBI Gene Expression Omnibus (GEO) database and are accessible through the accession number GEO: GSE329124. Additional data, materials, and related protocols will be available upon request from the corresponding author (Zongkui Wang) to comply with institutional ethics regulations.

ACKNOWLEDGMENTS

This work was supported by Science & Technology Department of Sichuan Province (2026NSFSC0545 and 2025ZDZX0043), CAMS Innovation Fund for Medical Sciences (CIFMS, 2021-I2M-1–042) and Scientific Research Project of Sichuan Medical Association (Q22009). In addition, we are grateful to Yang Liu from the Biomedical Analysis Center, Army Medical University, for his technical support during the experiments.

AUTHOR CONTRIBUTIONS

D.L., C.L., Z.W., and J.H. conceived and designed the study, conducted animal experiments, analyzed and interpreted the data, and drafted the manuscript; T.C., P.J., L.M., and Z.X. conducted some animal studies; T.C., F.L., P.F., X.D., and Z.X. analyzed the data and interpreted the results; J.X. and L.C. provided statistical and graphic editing support; D.L., C.L., and Z.W. revised the manuscript. All authors have read and approved of the final manuscript.

DECLARATION OF INTERESTS

The authors declare that they have no conflict of interest.

STAR★METHODS

Detailed methods are provided in the online version of this paper and include the following:

- KEY RESOURCES TABLE
- EXPERIMENTAL MODEL AND STUDY PARTICIPANT DETAILS
  - Animals
  - Enteroid cultures
- METHOD DETAILS
  - Radiation protocol
  - IVIg treatment and experimental grouping
  - Tissue collection and immunostaining
  - TUNEL staining
  - Lineage tracing of $LGR5^+$ ISCs
  - Crypt isolation and enteroid culture
  - Western blotting
  - Transcriptome analyses
- QUANTIFICATION AND STATISTICAL ANALYSIS
  - Statistical analysis and Blinded Assessment

SUPPLEMENTAL INFORMATION

Supplemental information can be found online at https://doi.org/10.1016/j.stemcr.2026.102934.

Received: December 8, 2025
Revised: May 5, 2026
Accepted: May 6, 2026
Published: June 4, 2026

REFERENCES

Bensemmane, L., Squiban, C., Demarquay, C., Mathieu, N., Benderitter, M., Le Guen, B., Milliat, F., and Linard, C. (2021). The stromal vascular fraction mitigates radiation-induced gastrointestinal syndrome in mice. Stem Cell Res. Ther. *12*, 309. https://doi.org/10.1186/s13287-021-02373-y.

Bhutta, B.S., Fatima, R., and Aziz, M. (2023). Radiation Enteritis (StatPearls).

Bussel, J.B., Vander Haar, E.L., and Berkowitz, R.L. (2021). New developments in fetal and neonatal alloimmune thrombocytopenia. Am. J. Obstet. Gynecol. *225*, 120–127. https://doi.org/10.1016/j.ajog.2021.04.211.

Charlet, R., Sendid, B., Kaveri, S.V., Poulain, D., Bayry, J., and Jawhara, S. (2019). Intravenous Immunoglobulin Therapy Eliminates Candida albicans and Maintains Intestinal Homeostasis in a Murine Model of Dextran Sulfate Sodium-Induced Colitis. Int. J. Mol. Sci. *20*, 1473. https://doi.org/10.3390/ijms20061473.

Clevers, H. (2013). The intestinal crypt, a prototype stem cell compartment. Cell *154*, 274–284. https://doi.org/10.1016/j.cell.2013.07.004.

de Lau, W., Peng, W.C., Gros, P., and Clevers, H. (2014). The R-spondin/LGR5/Rnf43 module: regulator of Wnt signal strength. Genes Dev. *28*, 305–316. https://doi.org/10.1101/gad.235473.113.

Fan, J., Lin, B., Fan, M., Niu, T., Gao, F., Tan, B., and Du, X. (2022). Research progress on the mechanism of radiation enteritis. Front. Oncol. *12*, 888962. https://doi.org/10.3389/fonc.2022.888962.

Fu, G., Chen, S., Liang, L., Li, X., Tang, P., Rao, X., Pan, M., Xu, X., Li, Y., Yao, Y., et al. (2021). SIRT1 inhibitors mitigate radiation-induced GI syndrome by enhancing intestinal-stem-cell survival. Cancer Lett. *501*, 20–30. https://doi.org/10.1016/j.canlet.2020.12.034.

Gandle, C., Dhingra, S., and Agarwal, S. (2020). Radiation-Induced Enteritis. Clin. Gastroenterol. Hepatol. *18*, A39–A40. https://doi.org/10.1016/j.cgh.2018.11.060.

Ganigara, M., Sharma, C., and Bayry, J. (2021). Unraveling the mechanisms of IVIG immunotherapy in MIS-C. Cell Rep. Med. *2*, 100431. https://doi.org/10.1016/j.xcrm.2021.100431.

Gibbons, C.H., and Klein, C. (2021). IVIG and Small Fiber Neuropathy: The Ongoing Search for Evidence. Neurology *96*, 929–930. https://doi.org/10.1212/WNL.0000000000011921.

Gong, W., Guo, M., Han, Z., Wang, Y., Yang, P., Xu, C., Wang, Q., Du, L., Li, Q., Zhao, H., et al. (2016). Mesenchymal stem cells stimulate intestinal stem cells to repair radiation-induced intestinal injury. Cell Death Dis. *7*, e2387. https://doi.org/10.1038/cddis.2016.276.

Gregorieff, A., Liu, Y., Inanlou, M.R., Khomchuk, Y., and Wrana, J.L. (2015). Yap-dependent reprogramming of LGR5(+) stem cells drives intestinal regeneration and cancer. Nature *526*, 715–718. https://doi.org/10.1038/nature15382.

Gregorieff, A., Pinto, D., Begthel, H., Destrée, O., Kielman, M., and Clevers, H. (2005). Expression pattern of Wnt signaling components in the adult intestine. Gastroenterology *129*, 626–638. https://doi.org/10.1016/j.gastro.2005.06.007.

He, J., Jiang, P., Ma, L., Liu, F., Fu, P., Du, X., Xu, Z., Xu, J., Cheng, L., Wang, Z., et al. (2024). Intravenous immunoglobulin protects the integrity of the intestinal epithelial barrier and inhibits ferroptosis induced by radiation exposure by activating the mTOR pathway. Int. Immunopharmacol. *131*, 111908. https://doi.org/10.1016/j.intimp.2024.111908.

He, X.C., Zhang, J., Tong, W.G., Tawfik, O., Ross, J., Scoville, D.H., Tian, Q., Zeng, X., He, X., Wiedemann, L.M., et al. (2004). BMP signaling inhibits intestinal stem cell self-renewal through suppression of Wnt-beta-catenin signaling. Nat. Genet. *36*, 1117–1121. https://doi.org/10.1038/ng1430.

Howard, J.F., Jr., Utsugisawa, K., Benatar, M., Murai, H., Barohn, R.J., Illa, I., Jacob, S., Vissing, J., Burns, T.M., Kissel, J.T., et al. (2017). Safety and efficacy of eculizumab in anti-acetylcholine receptor antibody-positive refractory generalised myasthenia gravis (REGAIN): a phase 3, randomised, double-blind, placebo-controlled, multicentre study. Lancet Neurol. *16*, 976–986. https://doi.org/10.1016/S1474-4422(17)30369-1.

Hwang, S.Y., Deng, X., Byun, S., Lee, C., Lee, S.J., Suh, H., Zhang, J., Kang, Q., Zhang, T., Westover, K.D., et al. (2016). Direct Targeting of beta-Catenin by a Small Molecule Stimulates Proteasomal Degradation and Suppresses Oncogenic Wnt/beta-Catenin

Signaling. Cell Rep. *16*, 28–36. https://doi.org/10.1016/j.celrep.2016.05.071.

Jang, H., Kim, S., Kim, H., Oh, S.H., Kwak, S.Y., Joo, H.W., Lee, S.B., Jang, W.I., Park, S., and Shim, S. (2022). Metformin Protects the Intestinal Barrier by Activating Goblet Cell Maturation and Epithelial Proliferation in Radiation-Induced Enteropathy. Int. J. Mol. Sci. *23*, 5929. https://doi.org/10.3390/ijms23115929.

Jun, S., Jung, Y.S., Suh, H.N., Wang, W., Kim, M.J., Oh, Y.S., Lien, E.M., Shen, X., Matsumoto, Y., McCrea, P.D., et al. (2016). LIG4 mediates Wnt signalling-induced radioresistance. Nat. Commun. *7*, 10994. https://doi.org/10.1038/ncomms10994.

Karnam, A., Rambabu, N., Das, M., Bou-Jaoudeh, M., Delignat, S., Käsermann, F., Lacroix-Desmazes, S., Kaveri, S.V., and Bayry, J. (2020). Therapeutic normal IgG intravenous immunoglobulin activates Wnt-beta-catenin pathway in dendritic cells. Commun. Biol. *3*, 96. https://doi.org/10.1038/s42003-020-0825-4.

Kozicky, L.K., Menzies, S.C., Hotte, N., Madsen, K.L., and Sly, L.M. (2019). Intravenous immunoglobulin (IVIg) or IVIg-treated macrophages reduce DSS-induced colitis by inducing macrophage IL-10 production. Eur. J. Immunol. *49*, 1251–1268. https://doi.org/10.1002/eji.201848014.

Kwak, S.Y., Jang, W.I., Park, S., Cho, S.S., Lee, S.B., Kim, M.J., Park, S., Shim, S., and Jang, H. (2021). Metallothionein 2 activation by pravastatin reinforces epithelial integrity and ameliorates radiation-induced enteropathy. EBioMedicine *73*, 103641. https://doi.org/10.1016/j.ebiom.2021.103641.

Lee, Y.S., Kim, T.Y., Kim, Y., Lee, S.H., Kim, S., Kang, S.W., Yang, J.Y., Baek, I.J., Sung, Y.H., Park, Y.Y., et al. (2018). Microbiota-Derived Lactate Accelerates Intestinal Stem-Cell-Mediated Epithelial Development. Cell Host Microbe *24*, 833–846.e6. https://doi.org/10.1016/j.chom.2018.11.002.

Leibowitz, B.J., Zhao, G., Wei, L., Ruan, H., Epperly, M., Chen, L., Lu, X., Greenberger, J.S., Zhang, L., and Yu, J. (2021). Interferon b drives intestinal regeneration after radiation. Sci. Adv. *7*, eabi5253. https://doi.org/10.1126/sciadv.abi5253.

Levine, D.S., Fischer, S.H., Christie, D.L., Haggitt, R.C., and Ochs, H.D. (1992). Intravenous immunoglobulin therapy for active, extensive, and medically refractory idiopathic ulcerative or Crohn's colitis. Am. J. Gastroenterol. *87*, 91–100.

Lindemans, C.A., Calafiore, M., Mertelsmann, A.M., O'Connor, M.H., Dudakov, J.A., Jenq, R.R., Velardi, E., Young, L.F., Smith, O.M., Lawrence, G., et al. (2015). Interleukin-22 promotes intestinal-stem-cell-mediated epithelial regeneration. Nature *528*, 560–564. https://doi.org/10.1038/nature16460.

Loge, L., Florescu, C., Alves, A., and Menahem, B. (2020). Radiation enteritis: Diagnostic and therapeutic issues. J. Visc. Surg. *157*, 475–485. https://doi.org/10.1016/j.jviscsurg.2020.08.012.

Metcalfe, C., Kljavin, N.M., Ybarra, R., and de Sauvage, F.J. (2014). LGR5+ stem cells are indispensable for radiation-induced intestinal regeneration. Cell Stem Cell *14*, 149–159. https://doi.org/10.1016/j.stem.2013.11.008.

Nusse, R., and Clevers, H. (2017). Wnt/beta-Catenin Signaling, Disease, and Emerging Therapeutic Modalities. Cell *169*, 985–999. https://doi.org/10.1016/j.cell.2017.05.016.

Rao, X., Tang, P., Li, Y., Fu, G., Chen, S., Xu, X., Zhou, Y., Li, X., Zhang, L., Mo, S., et al. (2021). CBP/P300 Inhibitors Mitigate Radiation-Induced GI Syndrome by Promoting Intestinal Stem Cell-Mediated Crypt Regeneration. Int. J. Radiat. Oncol. Biol. Phys. *110*, 1210–1221. https://doi.org/10.1016/j.ijrobp.2021.01.046.

Ren, L., Xie, D., Li, P., Qu, X., Zhang, X., Xing, Y., Zhou, P., Bo, X., Zhou, Z., and Wang, S. (2016). Radiation protective effects of baclofen predicted by a computational drug repurposing strategy. Pharmacol. Res. *113*, 475–483. https://doi.org/10.1016/j.phrs.2016.09.024.

Reynolds, A., Wharton, N., Parris, A., Mitchell, E., Sobolewski, A., Kam, C., Bigwood, L., El Hadi, A., Münsterberg, A., Lewis, M., et al. (2014). Canonical Wnt signals combined with suppressed TGFbeta/BMP pathways promote renewal of the native human colonic epithelium. Gut *63*, 610–621. https://doi.org/10.1136/gutjnl-2012-304067.

Saha, S., Aranda, E., Hayakawa, Y., Bhanja, P., Atay, S., Brodin, N.P., Li, J., Asfaha, S., Liu, L., Tailor, Y., et al. (2016). Macrophage-derived extracellular vesicle-packaged WNTs rescue intestinal stem cells and enhance survival after radiation injury. Nat. Commun. *7*, 13096. https://doi.org/10.1038/ncomms13096.

Schneider, C.A., Rasband, W.S., and Eliceiri, K.W. (2012). NIH Image to ImageJ: 25 years of image analysis. Nat. Methods *9*, 671–675. https://doi.org/10.1038/nmeth.2089.

Toullec, A., Buard, V., Rannou, E., Tarlet, G., Guipaud, O., Robine, S., Iruela-Arispe, M.L., François, A., and Milliat, F. (2018). HIF-1alpha Deletion in the Endothelium, but Not in the Epithelium, Protects From Radiation-Induced Enteritis. Cell. Mol. Gastroenterol. Hepatol. *5*, 15–30. https://doi.org/10.1016/j.jcmgh.2017.08.001.

van der Flier, L.G., Haegebarth, A., Stange, D.E., van de Wetering, M., and Clevers, H. (2009). OLFM4 is a robust marker for stem cells in human intestine and marks a subset of colorectal cancer cells. Gastroenterology *137*, 15–17. https://doi.org/10.1053/j.gastro.2009.05.035.

Wang, W., Xue, C., and Mao, X. (2020). Radioprotective effects and mechanisms of animal, plant and microbial polysaccharides. Int. J. Biol. Macromol. *153*, 373–384. https://doi.org/10.1016/j.ijbiomac.2020.02.203.

Wang, Z., Wang, Q., Gong, L., Liu, T., Wang, P., Yuan, Z., and Wang, W. (2022). The NF-kappaB-Regulated miR-221/222/Syndecan-1 Axis and Intestinal Mucosal Barrier Function in Radiation Enteritis. Int. J. Radiat. Oncol. Biol. Phys. *113*, 166–176. https://doi.org/10.1016/j.ijrobp.2022.01.006.

Wang, Z., Xiao, H., Dong, J., Li, Y., Wang, B., Chen, Z., Zeng, X., Liu, J., Dong, Y., Ma, L., et al. (2023). Sexual dimorphism in gut microbiota dictates therapeutic efficacy of intravenous immunoglobulin on radiotherapy complications. J. Adv. Res. *46*, 123–133. https://doi.org/10.1016/j.jare.2022.06.002.

Willison, H.J., Jacobs, B.C., and van Doorn, P.A. (2016). Guillain-Barre syndrome. Lancet *388*, 717–727. https://doi.org/10.1016/S0140-6736(16)00339-1.

Xiao, H.W., Cui, M., Li, Y., Dong, J.L., Zhang, S.Q., Zhu, C.C., Jiang, M., Zhu, T., Wang, B., Wang, H.C., and Fan, S.J. (2020). Gut microbiota-derived indole 3-propionic acid protects against radiation toxicity via retaining acyl-CoA-binding protein. Microbiome *8*, 69. https://doi.org/10.1186/s40168-020-00845-6.

Yan, K.S., Janda, C.Y., Chang, J., Zheng, G.X.Y., Larkin, K.A., Luca, V.C., Chia, L.A., Mah, A.T., Han, A., Terry, J.M., et al. (2017). Non-equivalence of Wnt and R-spondin ligands during LGR5(+) intestinal stem-cell self-renewal. Nature *545*, 238–242. https://doi.org/10.1038/nature22313.

Yang, L., Yang, H., Chu, Y., Song, Y., Ding, L., Zhu, B., Zhai, W., Wang, X., Kuang, Y., Ren, F., et al. (2021). CREPT is required for murine stem cell maintenance during intestinal regeneration. Nat. Commun. *12*, 270. https://doi.org/10.1038/s41467-020-20636-9.

Zhu, X., Yang, M., Lin, Z., Mael, S.K., Li, Y., Zhang, L., Kong, Y., Zhang, Y., Ren, Y., Li, J., et al. (2022). REGgamma drives LGR5(+) stem cells to potentiate radiation induced intestinal regeneration. Sci. China Life Sci. *65*, 1608–1623. https://doi.org/10.1007/s11427-021-2018-7.

Zuercher, A.W., Spirig, R., Baz Morelli, A., and Käsermann, F. (2016). IVIG in autoimmune disease - Potential next generation biologics. Autoimmun. Rev. *15*, 781–785. https://doi.org/10.1016/j.autrev.2016.03.018.

STAR★METHODS

KEY RESOURCES TABLE

REAGENT or RESOURCE	SOURCE	IDENTIFIER
**Antibodies**		
Mouse monoclonal anti-β-Catenin	BD Biosciences	Cat#610153
Rabbit polyclonal anti-Chromogranin A	Proteintech	Cat#60135-1-1g
Rabbit polyclonal anti-LYSOZYME	Proteintech	Cat#15013-1-AP
Rabbit monoclonal anti-OLFM4	CST	Cat#39141S
Rabbit monoclonal anti-pGSK-3β	CST	Cat#9323T
Rabbit monoclonal anti-γH2AX	CST	Cat#9718T
Rabbit polyclonal anti-Ki67	Abcam	Cat#ab1667
Mouse monoclonal anti-8-OHdG	Abcam	Cat#ab48508
Rat monoclonal anti-BrdU	Abcam	Cat#ab6326
Rabbit monoclonal anti-Cleaved-caspase3	CST	Cat#9661S
Rabbit polyclonal anti-LGR5	ABGENT	Cat#AP2745d-ev
Rabbit monoclonal anti-FABP1	CST	Cat#13368T
Rabbit polyclonal anti-GSK3β	Proteintech	Cat#22104-1-AP
Rabbit polyclonal anti-GSDMD	Abcam	Cat#ab209845
Rabbit polyclonal anti-Cleaved GSDMD-N	Abcam	Cat#ab215203
Mouse monoclonal anti-β-ACTIN	Abclonal	Cat#AC004
Mouse monoclonal anti-GAPDH	Abclonal	Cat#AC033
Rabbit polyclonal anti-Histone-H3	Proteintech	Cat#17168-1-AP
**Chemicals, peptides, and recombinant proteins**		
MSAB (Wnt/β-catenin inhibitor)	MedChemExpress	HY-12069
**Critical commercial assays**		
TUNEL apoptosis assay kit	Beyotime	Cat#C1086
IntestiCult™ mouse enteroid growth medium	Stemcell Technologies	#06005
**Deposited data**		
Raw and analyzed data	this study	GSE329124
**Experimental models: Organisms/strains**		
Mouse: C57BL/6J, wild type	GemPharmatech (Nanjing, China)	N/A
Mouse: Lgr5-EGFP-IRES-creERT2	The Jackson Laboratory	JAX: 008875
Mouse: Rosa26-tdTomato	The Jackson Laboratory	JAX: 007914
**Software and algorithms**		
ImageJ	Schneider et al., 2012	https://imagej.nih.gov/ij/

(*Continued on next page*)

Continued

REAGENT or RESOURCE	SOURCE	IDENTIFIER
GraphPad Prism 8.0	GraphPad Software	https://www.graphpad.com/
DAVID online analysis system	NIAID, NIH	https://david.ncifcrf.gov/
R software	R Foundation	https://www.r-project.org/
**Other**		
X-Rad 320 irradiator	PXI (Connecticut, USA)	N/A
Nikon confocal microscope	Nikon	N/A
Olympus BX53 microscope	Olympus	N/A
ZEISS Axio Observer with Apotome3	ZEISS	N/A

EXPERIMENTAL MODEL AND STUDY PARTICIPANT DETAILS

Animals

C57BL/6J male mice aged 6–8 weeks (22–25 g) were purchased from GemPharmatech (Nanjing, China). *Lgr*-EGFP-IRES-creERT2 and Rosa26-tdTomato mice were obtained from Jackson Laboratory (Bar Harbor, ME, USA) and crossed to generate *Lgr*-EGFP-IRES-creERT2/Rosa26-tdTomato offspring for experiments. All mice were housed in a specific-pathogen-free (SPF) facility under a 12-h light/dark cycle with free access to food and water. Animal protocols were approved by the Ethics Committee of the Institute of Blood Transfusion, Chinese Academy of Medical Sciences and Peking Union Medical College (Approval No. 2022034).

Enteroid cultures

Enteroids were derived from the small intestinal crypts of male C57BL/6J mice (as described in crypt isolation and enteroid culture section below). The sex of the cells is male, consistent with the donor mice. Enteroids were cultured in Matrigel (Corning, Canada) and maintained in IntestiCult™ enteroid growth medium (Stemcell Technologies, Canada) at 37°C with 5% CO_2. The culture medium was replaced every 3 days.

METHOD DETAILS

A complete list of resources (including antibodies, reagents, mouse strains, and software) used in this study along with their sources, catalog numbers, and RRIDs is provided in the Key Resources Table.

Radiation protocol

Total abdominal irradiation (TAI) was performed using an X-Rad 320 irradiator (PXI, Connecticut, USA). Briefly, adult C57BL/6J mice were anesthetized using 1% pentobarbital sodium, and their abdomens (3 cm wide, from the xiphoid process of the sternum to the symphysis pubis) were irradiated with a single dose of 12 Gy X-ray at a dose rate of 75 cGy/min. Other parts were shielded from exposure with lead plates. Enteroids (small intestine-derived organoids) were cultured in IntestiCult^{TM} medium (Stemcell Technologies, Canada) for 72 h, followed by a single dose X-ray irradiation of 6 Gy at a dose rate of 2.5 Gy/min.

IVIg treatment and experimental grouping

IVIg preparation was provided by Shanghai RAAS Blood Products Co., Ltd. The content of human immunoglobulin G (IgG) is not less than 95%, with the remainder mainly consisting of trace amounts of IgA and IgM. Following TAI, IVIg was administered intravenously a dose of 500 mg/kg body weight, and then injections were repeated every three days. The experimental groups were as follows: control group (Ctrl), total abdominal irradiation + PBS group (TAI), abdominal irradiation + IVIg group (TAI/IVIg), and abdominal irradiation + IVIg + MSAB inhibitor group (TAI/IVIg/MSAB). MSAB was purchased from MedChemExpress (New Jersey, USA). After irradiation, enteroids were incubated with IVIg (2.5 mg/mL) or an equal volume of PBS. The experimental groups were as follows: control group (Ctrl), radiation + PBS group (Rad), and radiation + IVIg group (Rad/IVIg).

Tissue collection and immunostaining

Mice were sacrificed at the indicated time points during experiments, and the jejunum of each mouse was collected for all subsequent biological experiments and analyses. After fixation with 4% paraformaldehyde for at least 72 h, the intestines were dehydrated and embedded in paraffin or OCT compound to prepare sections. Paraffin slides were used for hematoxylin & eosin (HE) staining, immunohistochemistry (IHC) staining and immunofluorescent (IF) staining. In brief, rehydrated slides were incubated with antibodies against Ki67, OLFM4, LGR5, β-Catenin and BrdU at 4°C overnight. After washing, specific secondary antibody was incubated (IHC staining required the addition of DAB staining step), and finally the nucleus was stained. The slides were observed with a Nikon confocal microscope or an Olympus BX53 microscope.

TUNEL staining

Paraffin slides were deparaffinized and rehydrated and then repaired in an EDTA antigen retrieval buffer (pH 8.0). Briefly, a proteinase K solution was added, and the slides were incubated at 37°C for 30 min and placed in a wet box. After washing, slides were incubated in TdT reaction buffer at 37°C for 30-60 min. Then, the slides were washed again, followed by the addition of a streptavidin-FITC reagent and incubation at 37°C for 30 min in the dark. Finally, after washing slides, DAPI was added to stain the nuclei, and antifading mounting medium was added dropwise to mount the plate, which was observed and photographed under a fluorescence microscope.

Lineage tracing of LGR5$^+$ ISCs

To observe the protective effect of IVIg on ISCs, we crossed the *Lgr*-EGFP-IRES-creERT2 and tdTomato lines to obtain *Lgr*-EGFP-IRES-creERT2/Rosa26-tdTomato mice. At the age of 6-8 weeks, mice were used for experiments. One hour after irradiation, the mice were intraperitoneally injected with tamoxifen (2 mg/20 g body weight) to activate Cre recombinase. The mice were sacrificed at 24 h after TAI, and small intestines were collected to prepare frozen sections. *Lgr*-EGFP$^+$ and tdTomato$^+$ *Lgr*-derived cells were observed using a ZEISS Axio Observer with Apotome3.

Crypt isolation and enteroid culture

Small intestines (approximately 15 cm in length) from different mice were freshly isolated and washed with cold PBS, then opened longitudinally and cut into 5 cm pieces. The tissue segments were washed again with cold PBS and incubated in 5 mM EDTA supplemented with antibiotics for 40 min. After removing EDTA, the tissues were washed with PBS and shaken to release crypts and villi. The supernatant contents were filtered through a 70 μm cell strainer (BD Falcon) to remove villi. Crypts were counted and enriched by centrifugation (1000 g, 5 min, 4°C). Subsequently, the pellets of crypts were resuspended in Matrigel (Corning, Canada) and seeded into 96-well plates. After 15 min of incubation at 37°C, crypts were cultured with IntestiCult™ enteroid growth medium (Stemcell Technologies, Canada), and the medium was replaced every 3 days.

Western blotting

Protease and phosphatase inhibitors were preadded to RIP buffer, and samples were lysed on ice for 30 min. Protein concentration was determined using the BCA method with a kit from Thermo Scientific. Samples were separated on SDS-polyacrylamide gels, and then immunoblotting was performed. Membranes were incubated with 5% skim milk for 1 h, followed by an overnight incubation with the designated primary antibody at 4°C. The membrane was washed and then incubated with a membrane-bound secondary antibody for 1 h at room temperature. Finally, the membrane was rinsed 3 times in TBST, and an enhanced chemiluminescence solution (Millipore) was evenly applied. The chemiluminescence signal was recorded with a Tanon system.

Transcriptome analyses

Mice were sacrificed on Day 3 after irradiation, and jejunal tissues from mice were rapidly frozen in liquid nitrogen. Intestinal RNA was extracted using the TRIzol method, and transcriptome sequencing was performed by Shanghai Zhongke New Life Biotechnology Co., Ltd. The differentially expressed gene (DEG) screening, volcano mapping, and GSEA were performed using an online analysis system. The R mfuzz package was used to cluster genes with different tendencies. In addition, KEGG and GO analyses were performed using the DAVID online analysis system, and the data were visualized using the ggplot package in R.

QUANTIFICATION AND STATISTICAL ANALYSIS

Statistical analysis and Blinded Assessment

Blinded Assessment: All organoid quantifications (survival and budding efficiency) were performed in a blinded manner by two independent researchers unaware of group assignments. Each experiment was independently repeated at least three times. Sample size (n) is provided in figure legends; n represents the number of independent biological replicates. Data were presented as mean ± SD. All statistical analyses were performed using GraphPad Prism 8.0. Comparisons between two groups were performed using unpaired, two-tailed Student's t-test. For comparisons involving more than two groups, one-way analysis of variance (ANOVA) followed by Tukey's post hoc test for multiple comparisons was used. Asterisks indicate statistical significance (*ns*: not significant; *$p < 0.05$; **$p < 0.01$; ***$p < 0.001$). p value < 0.05 was considered statistically significant.

Stem Cell Reports, Volume *21*

Supplemental Information

Intravenous immunoglobulin enhances intestinal stem cell regeneration to mitigate radiation-induced enteritis via promoting β-catenin nuclear translocation

Jia He, Tiancheng Chu, Ping Fu, Peng Jiang, Li Ma, Fengjuan Liu, Xi Du, Zhenni Xu, Jun Xu, Lu Cheng, Changqing Li, Dengqun Liu, and Zongkui Wang

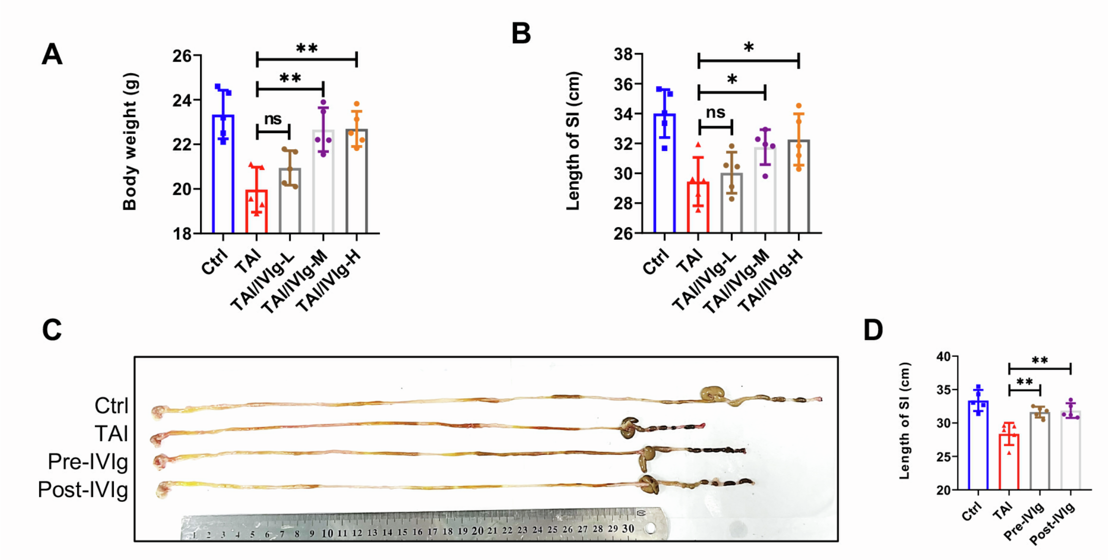

Figure S1. Effects of different IVIg concentrations and treatment timing on TAI mice. (A) Effects of different IVIg concentrations on body weight of TAI mice. (B) Effects of different IVIg concentrations on small intestinal length of TAI mice. (C) Effects of IVIg pretreatment and treatment on mouse intestinal tissue. (D) Statistical analysis of intestinal length. n = 5 mice. * $p < 0.05$, ** $p < 0.01$, *** $p < 0.001$, ns = not significant.

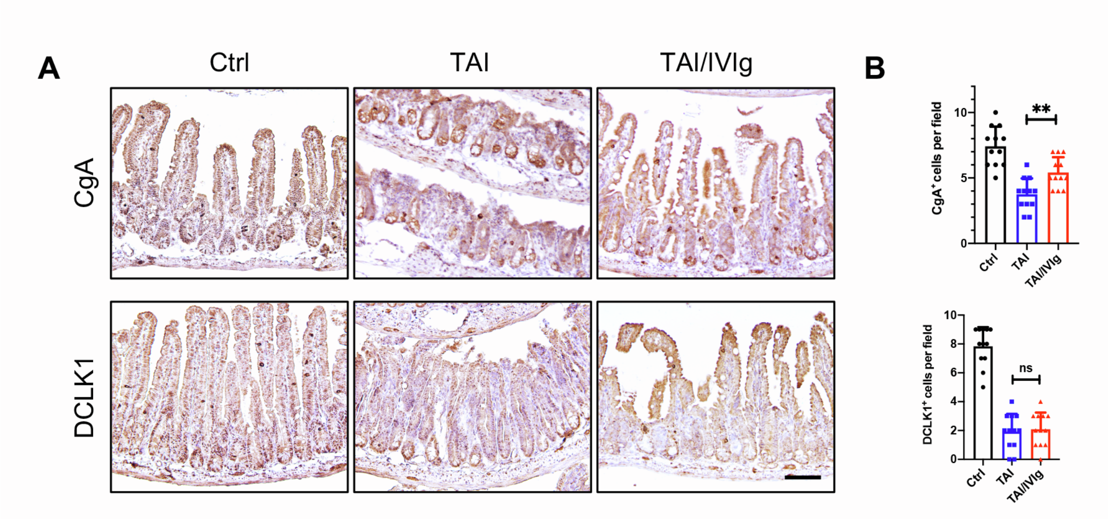

Figure S2. Effects of IVIg on other epithelial cells after TAI induction of RIE. (A) Representative images of CgA^{+} cells of small intestines (Bar = 100 μm). (B) Statistical analysis of CgA^{+} cells, n = 12 crypts. ** $p < 0.01$, ns = not significant.

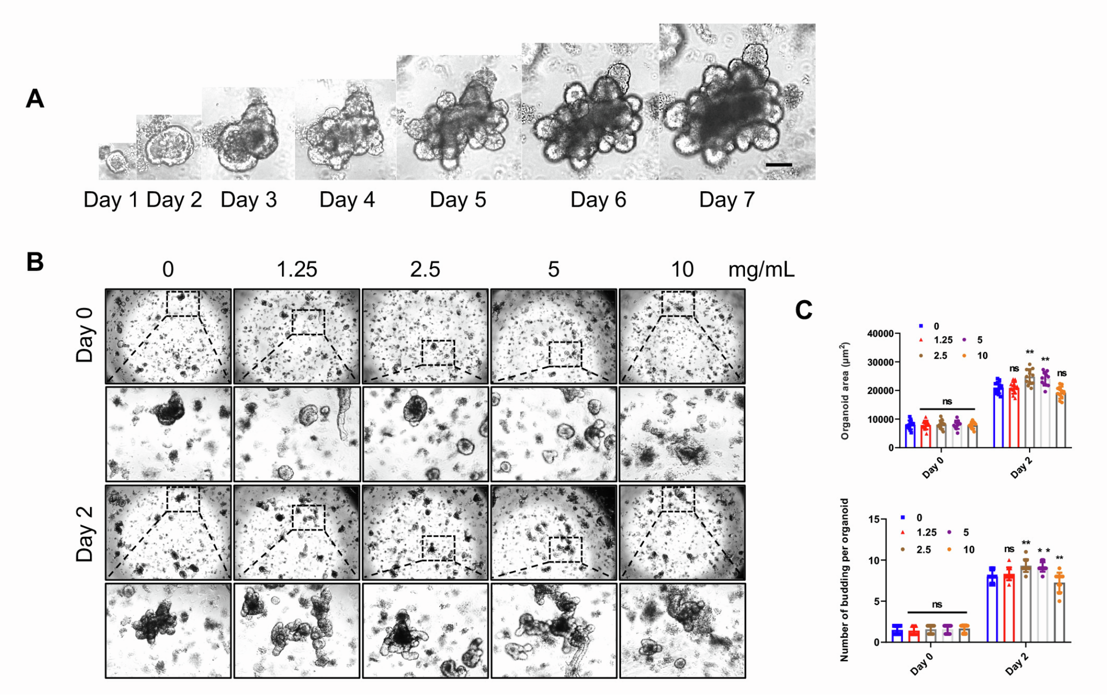

Figure S3. IVIg promoted the growth of healthy enteroids. (A) Morphological images of healthy intestinal enteroids under microscopy (Bar = 100 μm). (B) Microscopic images of intestinal enteroids after treatment with IVIg (Bar = 500 μm). (C) Quantitative analysis of the area and budding number per intestinal enteroid, n = 12 enteroids. ** $p < 0.01$, ns = not significant.

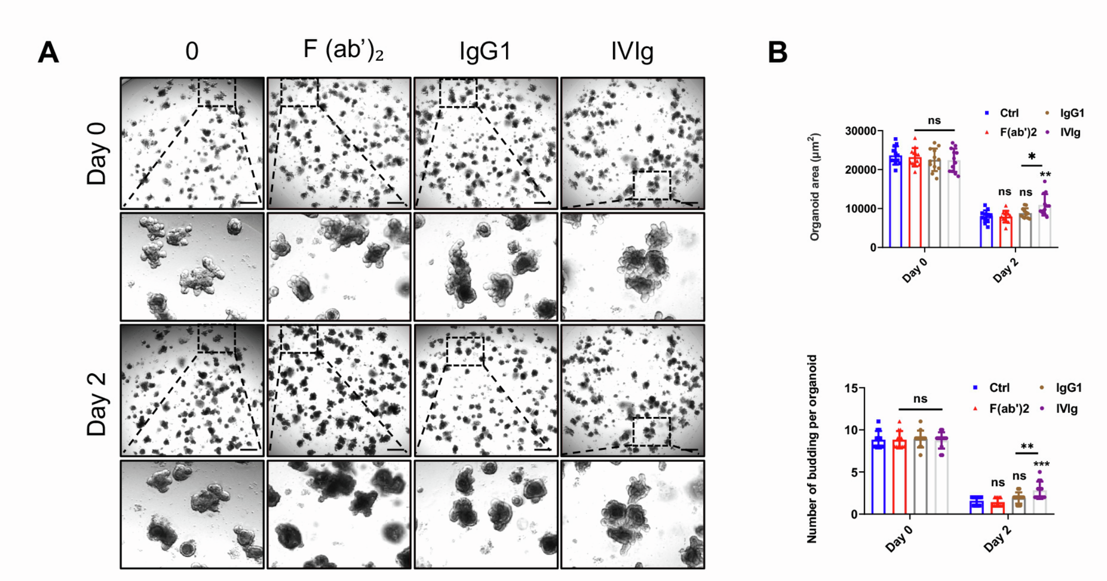

Figure S4. Effects of IVIg and its fragments on irradiated intestinal enteroids. (A) Representative microscopic images of irradiated intestinal enteroids treated with F(ab')$_2$, IgG1 or IVIg (Bar = 500 μm). (B) Quantitative analysis of the area and budding number per intestinal enteroid, n = 12 enteroids. * $p < 0.05$, ** $p < 0.01$, *** $p < 0.001$, ns = not significant.

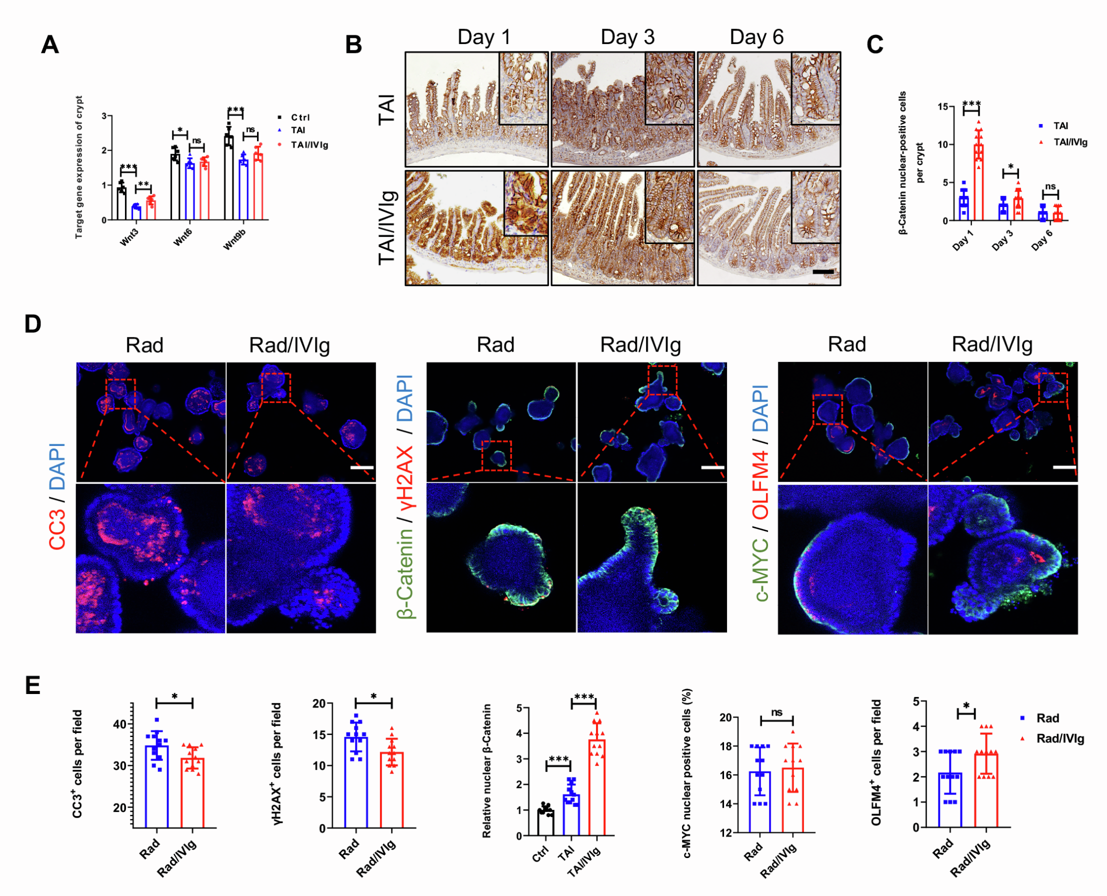

Figure S5. IVIg activates Wnt/β-Catenin signaling and alleviates radiation injury in intestinal crypts and organoids. (A) Changes in major *Wnt* subtypes in intestinal crypts of TAI mice after IVIg treatment. (B) Nuclear translocation of β-Catenin in mouse intestinal crypts shown by IHC. (C) Quantitative analysis of β-Catenin nuclear translocation. (D) Representative immunofluorescence images of Cleaved caspase-3, γ-H2AX, β-Catenin, OLFM4 and c-MYC and in enteroid. (E) Quantitative analysis of the markers shown in (D). * $p < 0.05$, ** $p < 0.01$, *** $p < 0.001$, ns = not significant.

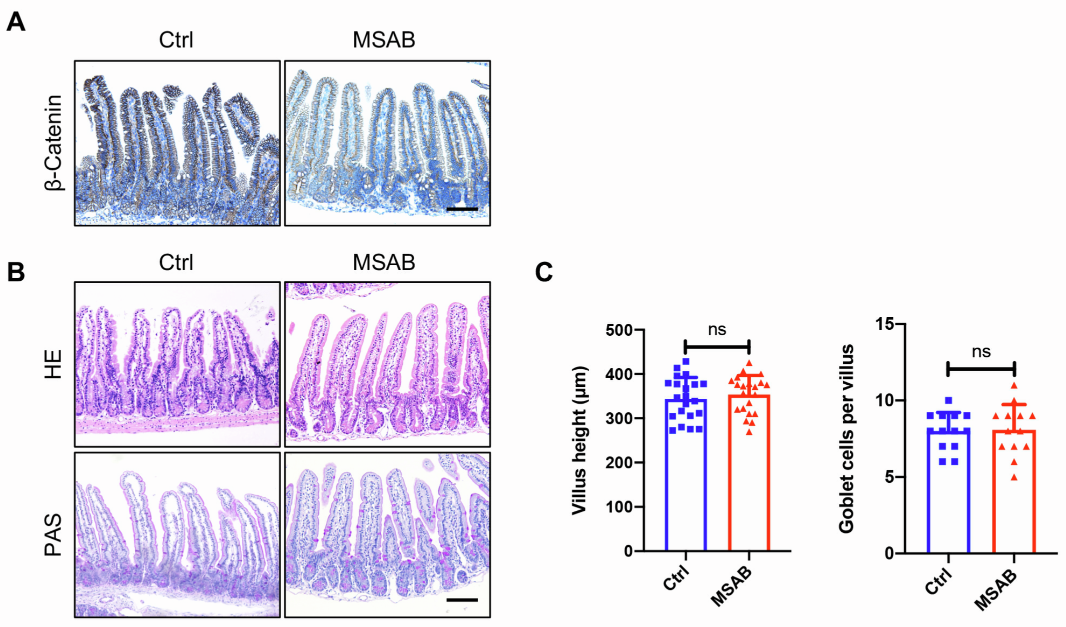

Figure S6. MSAB did not affect the intestine in short term. (A) Representative β-Catenin IHC-stained images of the small intestines (Bar = 100 μm). (B) Representative HE and PAS staining of the small intestines (Bar = 100 μm). (C) Statistical analysis of the villus heights and PAS$^+$ cells, n > 10 villi. ns = not significant.

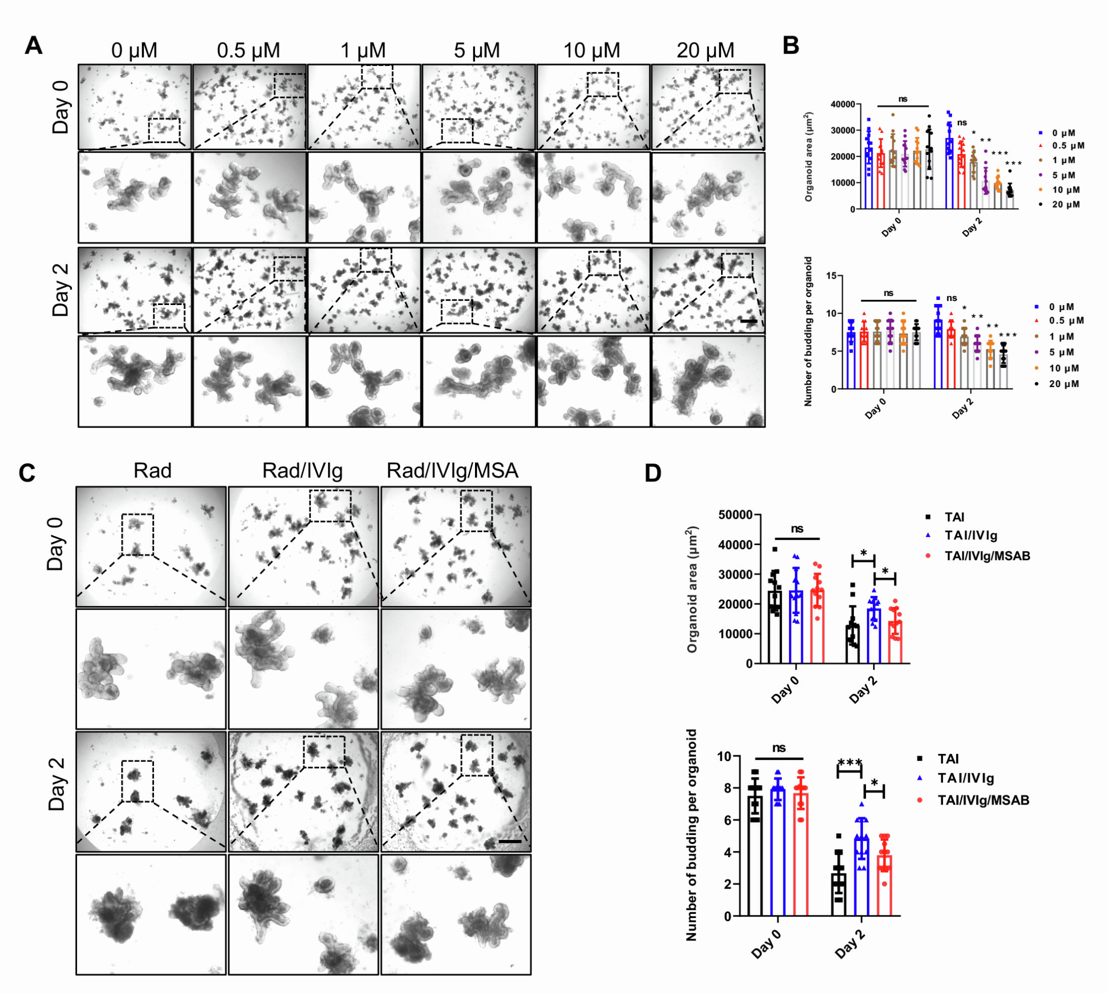

Figure S7. Blocking of the β-Catenin pathway eliminates the protective effect of IVIg on irradiation-damaged enteroids. (A) Microscopic images of mature enteroids after treatment with MSAB (Bar = 500 μm). (B) Quantitative analysis of enteroids area and budding number per enteroid, n > 10 crypts. (C) Microscopic images of enteroids after 6 Gy X-ray irradiation (scale bar = 500 μm). (D) Quantitative analysis of enteroids area and budding number per enteroid, n = 12 enteroids. * $p < 0.05$, ** $p < 0.01$, *** $p < 0.001$, ns = not significant.

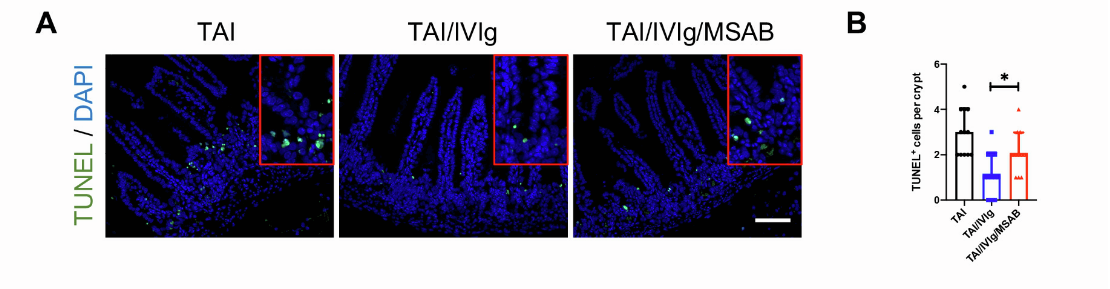

Figure S8. Blocking of the β-Catenin pathway eliminates the protective effect of IVIg against crypt apoptosis in TAI mice. (A) Representative TUNEL staining of small intestines (Bar = 100 μm). (B) Statistical analysis of TUNEL^{+} cells, n > 10 crypts. * $p < 0.05$.
